# Excessive self-grooming, gene dysregulation and imbalance between the striosome and matrix compartments in the striatum of *Shank3* mutant mice

**DOI:** 10.3389/fnmol.2023.1139118

**Published:** 2023-03-16

**Authors:** Allain-Thibeault Ferhat, Elisabeth Verpy, Anne Biton, Benoît Forget, Fabrice De Chaumont, Florian Mueller, Anne-Marie Le Sourd, Sabrina Coqueran, Julien Schmitt, Christelle Rochefort, Laure Rondi-Reig, Aziliz Leboucher, Anne Boland, Bertrand Fin, Jean-François Deleuze, Tobias M. Boeckers, Elodie Ey, Thomas Bourgeron

**Affiliations:** ^1^Génétique Humaine et Fonctions Cognitives, Institut Pasteur, CNRS UMR 3571, IUF, Université Paris Cité, Paris, France; ^2^Department of Neuroscience, Columbia University Irving Medical Center, New York, NY, United States; ^3^Zuckerman Mind Brain Behavior Institute, Columbia University, New York, NY, United States; ^4^Bioinformatics and Biostatistics Hub, Institut Pasteur, Université Paris Cité, Paris, France; ^5^Imagerie et Modélisation, Institut Pasteur, CNRS UMR 3691, Paris, France; ^6^Cerebellum Navigation and Memory Team, Institut de Biologie Paris Seine, Neurosciences Paris Seine, CNRS UMR 8246, Inserm UMR-S 1130, Sorbonne Université, Paris, France; ^7^Centre National de Recherche en Génomique Humaine, CEA, Université Paris-Saclay, Evry, France; ^8^Centre d’Étude du Polymorphisme Humain, Paris, France; ^9^Institute of Anatomy and Cell Biology, Ulm University, Ulm, Germany; ^10^Deutsches Zentrum für Neurodegenerative Erkrankungen, Ulm, Germany; ^11^Institut de Génétique et de Biologie Moléculaire et Cellulaire, CNRS UMR 7104, Inserm UMR-S 1258, Université de Strasbourg, Illkirch-Graffenstaden, France

**Keywords:** autism, *Shank3*, mouse model, stereotyped behavior, striatum compartmentation, GAD65, striosomes, matrix

## Abstract

Autism is characterized by atypical social communication and stereotyped behaviors. Mutations in the gene encoding the synaptic scaffolding protein SHANK3 are detected in 1–2% of patients with autism and intellectual disability, but the mechanisms underpinning the symptoms remain largely unknown. Here, we characterized the behavior of *Shank3*^Δ11/Δ11^ mice from 3 to 12 months of age. We observed decreased locomotor activity, increased stereotyped self-grooming and modification of socio-sexual interaction compared to wild-type littermates. We then used RNAseq on four brain regions of the same animals to identify differentially expressed genes (DEGs). DEGs were identified mainly in the striatum and were associated with synaptic transmission (e.g., *Grm2, Dlgap1*), G-protein-signaling pathways (e.g., *Gnal*, *Prkcg1*, *and Camk2g*), as well as excitation/inhibition balance (e.g., *Gad2*). Downregulated and upregulated genes were enriched in the gene clusters of medium-sized spiny neurons expressing the dopamine 1 (D1-MSN) and the dopamine 2 receptor (D2-MSN), respectively. Several DEGs (*Cnr1*, *Gnal*, *Gad2*, *and Drd4*) were reported as striosome markers. By studying the distribution of the glutamate decarboxylase GAD65, encoded by *Gad2*, we showed that the striosome compartment of *Shank3*^Δ11/Δ11^ mice was enlarged and displayed much higher expression of GAD65 compared to wild-type mice. Altogether, these results indicate altered gene expression in the striatum of *Shank3*-deficient mice and strongly suggest, for the first time, that the excessive self-grooming of these mice is related to an imbalance in the striatal striosome and matrix compartments.

## Introduction

Autism is a neurodevelopmental condition characterized by atypical social communication and interactions associated with stereotyped behaviors and restricted interests. More than 200 genes have been robustly associated with autism pointing at biological pathways such as chromatin remodeling, protein translation and synaptic function (Leblond et al., 2021). Among these genes, *SHANK3* (SH3 and multiple ankyrin repeat domain 3) codes for a scaffolding protein located at the postsynaptic density (PSD) of glutamatergic synapses where it interacts with other scaffolding proteins, cytoskeletal proteins, glutamate receptors and signaling molecules (Monteiro and Feng, 2017).

Heterozygous *de novo* mutations deleting *SHANK3* on chromosome 22q13.3 or affecting its coding region are associated with Phelan-McDermid syndrome (Phelan and McDermid, 2012) and observed in 1–2% of patients with both autism and intellectual disability, making this gene a major cause of neurodevelopmental disorder (Durand et al., 2007). Remarkably, a relatively high proportion of patients (>60%) carrying *SHANK3* mutations display regression (i.e., substantial loss of language and social skills) during adolescence and adulthood (Rubeis et al., 2018; Kohlenberg et al., 2020).

Through the use of multiple intragenic promoters and alternative splicing, several SHANK3 protein isoforms are differentially expressed according to developmental stage or brain region (Wang et al., 2014). Numerous *Shank3* mutant mice have been generated (for reviews see Ferhat et al., 2017; Monteiro and Feng, 2017). Most of them bear deletion of specific exons still allowing the expression of some isoforms (Wang et al., 2014; Drapeau et al., 2018). The behavioral deficits reported for *Shank3* mutant mice depend on the strains and experimental conditions. However, the presence of stereotyped-like behaviors, such as excessive self-grooming, was reported in all *Shank3* mutant mice and could be reminiscent of the stereotyped behaviors observed in patients with autism (Peça et al., 2011; Wang et al., 2011; Monteiro and Feng, 2017). Stereotyped behaviors are frequently associated with anomalies of the striatum (Kalueff et al., 2016), a region that coordinates multiple aspects of cognition, including action planning, decision-making, motivation, reinforcement, and reward perception as well as motor control. Several studies identified anomalies in the striatum of *Shank3* mutant mice at the anatomical (e.g., larger volume), circuit (e.g., disruption of indirect pathway of basal ganglia) and cellular (e.g., decreased striatal expression of GluN2B, a NMDA receptor subunit) levels (Peixoto et al., 2016; Reim et al., 2017; Wang et al., 2017; Bey et al., 2018; Schoen et al., 2019; Jin et al., 2021). The biological pathways underpinning the observed abnormalities remain however largely unknown.

In the present work, we first conducted a thorough longitudinal behavioral characterization of *Shank3*^∆11/Δ11^ mice, carrying a deletion of exon 11 of *Shank3* (supplementary information of Schmeisser et al., 2012), at 3, 8, and 12 months of age, focusing on social interactions, locomotion and stereotyped behaviors. We then compared gene expression in four brain structures - cortex, hippocampus, cerebellum and striatum - in *Shank3^+/+^* and *Shank3*^Δ11/Δ11^ male littermates characterized in the behavioral study. Compared to the other brain regions, the striatum appeared to be highly sensitive to the loss of SHANK3. We also identified a correlation between the level of self-grooming and an increased striatal expression of several genes, including *Gad2* encoding GAD65, which catalyzes the transformation of glutamate into γ-aminobutyric acid (GABA) at the synapse. Finally, using immunofluorescence on striatum sections at 12 months, we found that, compared to wild-type littermates, the *Shank3*^Δ11/Δ11^ mice displayed an enlargement of the compartment formed by striosomes/patches which also markedly over-expressed GAD65. These alterations of the striosomal compartment were also found in the *Shank3*^Δ4–22/Δ4-22^ mouse (deletion from exon 4 to 22) with a complete lack of all SHANK3 isoforms. Altogether our results point toward a correlation between excessive self-grooming and signaling imbalance between the striosome and matrix compartments of the striatum in SHANK3-deficient mice.

## Material and methods

### Animals

*Shank3*^∆11/∆11^ mice were generated by Genoway (Lyon, FRANCE) using 129S1/SVImJ ES cells. The *Shank3* mutation, deletion of exon 11, was then transferred onto a C57BL/6 J background with more than 15 backcrosses (supplementary information of Schmeisser et al., 2012). In this model, some protein isoforms are still expressed (supplementary information of Schmeisser et al., 2012). Mice were weaned at 22 ± 1 days of age and housed in same-sex mixed-genotype groups of three to five animals unless otherwise specified. They were housed under constant temperature (22 ± 1°C) with a 12:12 light/dark cycle (light on from 07:00 AM to 07:00 PM) and food and water were provided *ad libitum*. The experimenters were blind to the genotype of the tested animals for data collection and analyzes. We bred *Shank3*^∆11/+^ males and females to obtain *Shank3*^+/+^, *Shank3*^+/∆11^ and *Shank3*^∆11/∆11^ littermates. For behavioral tests, we used three cohorts (including both males and females) that we analyzed separately. Cohort 1 involved 44 males (13 *Shank3*^+/+^, 19 *Shank3*^+/∆11^, and 12 *Shank3*^∆11/∆11^) and 38 females (10 *Shank3*^+/+^, 16 *Shank3*^+/∆11^, and 12 *Shank3*^∆11/∆11^). Mice from Cohort 1 were evaluated at Institut Pasteur at 3 months of age -the standard age for behavioral testing- for dark–light, Y-maze, open field tests, self-grooming observation, 3-chambered test, free social interactions, as well as at 8 and 12 months of age for open field test, self-grooming and free social interaction. After the tests at 3 months of age, males were housed individually, while females remained group-housed. This difference of housing conditions between males and females was related to the increased aggressivity of males (C57BL/6 J background) after sexual experience with females in socio-sexual interactions at 3 months of age. Cohort 2 involved 42 males (15 *Shank3^+/+^*, 14 *Shank3*^+/∆11^, and 13 *Shank3*^∆11/∆11^) and 22 females (4 *Shank3^+/+^*, 11 *Shank3*^+/∆11^, and 7 *Shank3*^∆11/∆11^). Male and female mice from Cohort 2 were evaluated at Institut Pasteur at 3 months of age for self-grooming observation, open field, elevated plus maze, and dark–light anxiety tests. Cohort 3 involved 14 *Shank3*^+/+^ and 7 *Shank3*^∆11/∆11^ males that were bred at Institut Pasteur and sent at 2–2.5 months to Institut Biologie Paris Seine (IBPS). They were subjected to the open field, water maze and starmaze tests.

*Shank3*^∆4–22/∆4–22^ mice (Drapeau et al., 2018), with a C57BL/6 N background, were obtained from The Jackson Laboratory Repository.

### Behavioral tests

#### General health

At weaning, we measured weight and observed hind limb clasping as well as the physical aspect (fur, injury, or malformation) of the mice. At 3 and 12 months of age in Cohort 1, we again measured weight and noted the physical aspect of all mice.

#### Dark/light anxiety-like test

The mouse was left to freely explore a test cage separated into two compartments connected by a small door (5 × 5 cm; dark side: 3 lux; light side: 1300 lux obtained through a desk lamp directly above the compartment) for 5 min. The latency to enter the dark compartment and the time spent in each compartment were measured (Ethovision, Noldus Information Technologies, Wageningen, Netherlands). More anxious mice are expected to spend shorter time in the light compartment and do less transitions between the compartments.

#### Y-maze

The animal (Cohort 1) was allowed to freely explore a y-maze during 5 min. The number of entrances as well as the sequence of entrances into each arm were manually noted by an experimented scientist. The sequence of correct alternations, i.e., when the subject does not go back to the previous arm visited, are used to determine the working memory.

#### Elevated plus-maze anxiety-like test

The animal (Cohort 2) was allowed to freely explore the setup for 10 min. The elevated plus-maze (four arms of 7 cm by 30 cm, 50 cm above the floor) consisted of two open arms (no walls) and two closed arms (with walls), all connected by a neutral zone in the center (100 lux). We measured the time spent in the open and closed arms, as well as the number of transitions using Ethovision (Noldus Information Technologies, Wageningen, Netherlands). We interpret that the time spent in the closed arms is positively correlated with the anxiety level of the mice.

#### Locomotion and exploratory test in the open field

The mouse (Cohorts 1 and 2) was allowed to freely explore for 30 min a round open field arena of 1 m in diameter (100 lux in the center of the arena). We recorded the total distance traveled (Ethovision, Noldus Information Technology, Wageningen, Netherlands). Spontaneous locomotor activity of Cohort 3 was quantified in an arena made of gray perspex (45 × 45 cm) surrounded by red Plexiglas walls (30 cm height). Mice were first positioned in the center of the arena and were allowed to freely explore for 10 min. Data acquisition was performed at a frequency of 25 Hz using the SMART® video recording system and tracking software and traveled distance was computed using NAT (Navigation Analysis Tool), a custom Matlab-based software (Jarlier et al., 2013).

#### Observation of stereotyped behavior

Mice were individually placed in a new test cage (Plexiglas, 50 × 25 × 30 cm; 100 lux; clean sawdust bedding) in a soundproof chamber. After 10 min habituation, we recorded their behavior for 10 min (camera Logitech C920). We manually scored the time spent self-grooming and digging (The Observer, Noldus Information Technology). When scoring self-grooming, we did not take into account the scratching behavior. The total duration and the average event duration of each behavioral category were calculated.

#### Occupant/New-comer social test with ultrasonic vocalization recording

The tested mouse was isolated socially for 3 days (females) or 3 weeks (males) to increase motivation for affiliative social contacts (Ferhat et al., 2016). From this test on at 3 months of age, males remained singly housed. After 20 min of habituation for the tested mouse (the occupant) to the test cage (Plexiglas, 50 × 25 × 30 cm; 100 lux; clean sawdust bedding) in a soundproof chamber, an unfamiliar age- and sex-matched C57BL/6 J mouse (new-comer, NC; Charles River Laboratories, France) was introduced. The two mice were allowed to freely interact for 4 min. Social interactions (video camera Logitech C920, 30 fps) were semi-automatically analyzed using the 2D tracking module Mice Profiler from the ICY platform (de Chaumont et al., 2012a,b) (Institut Pasteur, Paris, France). We quantified, for both the occupant and the new-comer, the time spent in contact, the types of contact (oral-oral contact, oro-genital contact), the “approach then escape” sequences, the follow behavior, and the time spent in the vision field of the other one (Ferhat et al., 2015). At the same time, ultrasonic vocalizations (USV) were recorded (Condenser ultrasound microphone Polaroid/CMPA, UltraSoundGate 416–200, Avisoft Bioacoustics, Glienicke, Germany; sampling frequency: 300 kHz; FFT-length: 1024 points; 16-bit format). Vocalization files were analyzed automatically using the vocalization analysis plugin from LMT USV Toolbox (de Chaumont et al., 2021).

#### Male behavior in presence of an estrous female

The tested male was placed in the presence of a female for 48 h. Then, the male was isolated again for 1 day. During the test, the male was placed in the test cage (Plexiglas, 50 × 25 × 30 cm; 100 lux; with clean sawdust bedding) during 10 min for habituation (Ferhat et al., 2015). After this period, an unknown C57BL/6 J female in estrus (tested through vaginal smears in the morning) was introduced into the test cage for 4 min. Both mice were allowed to freely interact. Social interactions as well as USV were recorded, as described above.

#### Three-chambered social test

A Plexiglas cage was divided in three connected compartments (side compartments: 150 lux; central compartment: 140 lux) as previously described (Nadler et al., 2004). Both side compartments contained an empty wire cup. First, the tested mouse was allowed to freely explore the setting, with doors open for 10 min (phase 1) for habituation. Then, the mouse was restricted in the central compartment, while an unfamiliar C57BL/6 J mouse of the same sex (stranger 1) was placed under one of the cups (sides alternated between each mouse). The tested mouse was then allowed to explore the apparatus for 10 min (phase 2). In all phases, the time spent in each compartment and the number of transitions between compartments were automatically recorded. The time spent in contact with the cup containing the mouse (stranger 1) and the time spent in contact with the empty cup were manually measured in phase 2 to evaluate social interest.

#### Water maze with visible platform

Mice were trained in a circular water tank (150 cm in diameter, 40 cm high) to swim toward a visible platform (12 cm in diameter, 1 cm under the water surface) marked with an object (10 cm above the water surface), as previously described (Burguiere et al., 2005; Lefort et al., 2019). The platform was randomly placed at different locations across trials and the pool was surrounded by blue curtains to occlude extramaze cues. Training consisted in one training session per day, four trials per session, during 2 days. The starting position (North, East, West, or South) was randomly selected with each quadrant sampled once a day. At the beginning of each trial, the mouse was released at the starting point and made facing the inner wall. Then, it was given a maximum of 90 s to locate and climb onto the escape platform. If the mouse was unable to find the platform within the 90 s period, it was guided to the platform by the experimenter. In either case, the mouse was allowed to remain on the platform for 30 s. Data acquisition was performed at a frequency of 25 Hz using the SMART® video recording system and tracking software.

#### Starmaze

The starmaze consisted of five alleys radiating from the vertices of a central pentagonal ring. All the alleys were filled with water, and the water was made opaque with an inert nontoxic product (AccuScan OP 301, Brenntag). The maze was surrounded by a square black curtain with 2D and 3D patterns affixed to provide configurations of spatial cues. To avoid the possible use of a guidance strategy (i.e., animals could rely on the use of a single distal cue), cue was given in duplicate. White noise was used to cover all other sounds that the mice could have used to orientate themselves. To solve the task, animals had to swim to a trapezoidal platform (10 and 24 cm for top and bottom parallel sides, 11 cm of lateral sides) hidden 1 cm below the water surface and located 10 cm from the end of one alley. Departure and arrival points were always the same. All animals ran one session of five trials per day over 2 days using a 40-min inter-trial interval. If an animal did not locate the escape platform within 90 s, the experimenter placed the animal onto the platform for 30 s. During the protocol, one central alley and two peripheral alleys were blocked, forcing the mice to use the “left pathway.” Mice were tracked by using the Smart Software (Bioseb, Vitrolles, France).

#### Behavioral statistics

All group data are represented as mean ± standard error of the mean (s.e.m.), as well as the individual points. All statistics were performed with R software (R Core Team (2020), R Foundation for Statistical Computing). In all behavioral tests, *Shank3*^+/Δ11^ mice were not significantly different from *Shank3*^+/+^ unless otherwise specified (see Supplementary Table S1). Given the limited sample size and the non-normality of the data, comparisons between genotypes were performed using non-parametric Mann–Whitney *U*-tests. We used the non-parametric Friedman test to examine the effect of age within each genotype. Between age points, post-hoc tests were performed using paired Wilcoxon signed-rank tests. If required, a Bonferroni correction for multiple testing was performed. Differences between groups were considered significant when *p* < 0.05.

### Transcriptome analysis

#### Tissue collection

Mice were killed during the light phase, specifically between 9:00 and 11:00 AM, by CO_2_ intoxication. The brain was removed and macro-dissected on ice (4°C) into HBSS solution by an experienced practitioner. After separation of the hemispheres, six brain structures were extracted: whole cortex, hippocampus, whole striatum, cerebellum, diencephalon, and brainstem. Samples were snap frozen in liquid nitrogen and stored at −80°C.

#### Total RNA extraction and sequencing

Total RNA was extracted from four over six brain structures (whole cortex, hippocampus, whole striatum, cerebellum) using the miRNeasyPlus Micro Kit (Qiagen), following the manufacturer’s instructions, including DNAse digestion. After first quality assessment using the Nanodrop spectrophotometer ND-1000 (Thermoscientific), the samples were analyzed by the CNRGH (Centre National de Recherche en Génomique Humaine, CEA, Evry, France). RNA integrity was assessed using the Bioanalyzer RNA 6000 Nano assay and 2,100 Bioanalyzer (Agilent Technologies). Then, an oriented mRNA sequencing was performed on samples with a RNA Integrity Number (RIN) larger than eight. Eight *Shank3*^∆11/∆11^ and 7 *Shank3*^+/+^ 12-month old mice were used for the differential expression analysis. For each of these mice, we studied four brain regions (cerebellum, cortex, hippocampus, and striatum), except for one in each group for which only three of the four brain regions were available. The description of the samples is available in Supplementary Table S5. The data were obtained from two batches of sequencing performed at 8 months of interval. For the first batch, RNA extractions were performed on the same day while for the second sequencing batch, RNAs were extracted on two different days. We refer to this variable containing three levels as the batch variable. Each sequencing run included two flow cells. The RNA-seq datasets presented in the study are deposited in the Sequence Read Archive (SRA) repository, accession number PRJNA934367.

#### Mapping and reference genome

The RNAseq reads were mapped onto the genome with the STAR aligner v2.5.3a (Dobin et al., 2013) in 2-pass mode to a masked version of the *Mus Musculus* GRCm38 genome. During a first round of the differential gene expression analysis, we observed enrichment of the DEGs in genes located on the same arm of chromosome 15 as *Shank3*. Differences in genetic backgrounds around the *Shank3^+/+^* (C57BL/6 J) and the *Shank3*^∆11/∆11^ alleles (129S1/SvImJ, from the ES cells used to generate the *Shank3*^*∆*11^ mutation followed by 15 backcrosses on C57BL/6 J) could affect the mapping by impacting the mapped reads on the remaining region of 129S1/SvImJ around *Shank3*. To avoid this bias, we masked the GRCm38 genome for variants of the 129S1/SvImJ mouse strain before mapping the sequencing reads. Variants were extracted from the VCF file provided by The Mouse Genome Project (Yalcin et al., 2012)^1^ and masked in the reference genome using the SNPsplit software (Krueger and Andrews, 2016).

#### Calling variants from the RNAseq data

To see whether differences between the C57BL/6 J and the 129S1/SvImj genomes in the region around *Shank3* could explain the larger number of DEGs detected in the vicinity of *Shank3*, we used the RNAseq data to identify single nucleotide polymorphisms (SNPs) in each mouse. We followed the Gatk Best Practices (Van der Auwera et al., 2013) workflow for SNP and indel calling on RNAseq data^2^ which includes the following steps: (i) map to the reference genome with STAR in multi-sample 2-pass mode to get the most sensitive novel junction discovery; (ii) add read groups, sorting, marking duplicates, and create index, using Picard’s tools^3^; (iii) split reads into exon segments (removing Ns but maintaining grouping information) and hard clipping sequences overhanging into the intronic regions, using the SplitNCigarReads Gatk tool; (iv) realign indels and recalibrate Base quality; (v) call variant with HaplotypeCaller, and finally filter the variants with VariantFiltration. The last step was adapted to our project where several samples coming from the same mouse were available. HaplotypeCaller (with parameter -ERC BP_RESOLUTION) was called for each mouse individually using as input the processed BAM files coming from different brain tissues of the same mouse. Mice with the same *Shank3* status were then genotyped together by inputting the GVCF files to the Gatk tool GenotypeGVCFs. *Shank3*^∆11/∆11^- and *Shank3*^+/+^-specific VCF files were finally combined with the Gatk tool CombineVariants, which provides allele frequency and specificity of the variants to *Shank3*^∆11/∆11^ or *Shank3*^+/+^ mice populations. The resulting VCF file was filtered using the Gatk tool VariantFiltration using the parameters recommended in the Gatk workflow.

#### Differential expression analysis

The 18,194 genes with at least one count-per-million (CPM) in two samples were selected. The samples flagged by the QC Analyzer were filtered out. Multidimensional scaling plots showed separation of the samples according to brain regions, batches, and flow cells (Supplementary Figure S13). Differential gene expression analysis was performed with limma-voom v3.34.8 (Law et al., 2014), and the version of *voom* using sample-quality weights (Liu et al., 2015) (function *voomWithQualityWeights*) in order to take into account the sample heterogeneity observed within and across brain regions (Supplementary Figure S14). The Trimmed Mean of *M*-values (TMM) method was used to calculate normalization factors between samples. Three factors were included in the design matrix: the batch, the flow cell, and the brain tissue and *Shank3* status combined into one factor of eight levels. Since we were making comparisons both within and between mice, we treated the mouse as a random effect to adjust for baseline differences between subjects. To do so, the mouse was used as a blocking factor and the correlation between measurements made on the same mouse was computed using the function *duplicateCorrelation* and was input into the linear model fit, as suggested in the section “Multi-level Experiments” of the limma user guide. For each contrast of interest, the linear model was fitted for each gene using the function *lmFit*, and empirical Bayes smoothing was applied to the standard errors using the function *eBayes* with robust mode set to TRUE.

Gene-set over-representation analyses were performed using Fisher’s hypergeometric tests. Plots were made using the R packages ggplot2 (Wickham, 2009), upsetR (Conway et al., 2017), and ggbio (Yin et al., 2012).

### Gene set and protein–protein interaction network analysis

#### Gene set analysis

Gene set over-representation analyses (ORA) were performed with the egsea.ora function available in the Bioconductor R package EGSEA (Ensemble of Gene Set Enrichment Analyses) v1.6.1 (Alhamdoosh et al., 2017) without considering the genes around the *Shank3* gene. All collections of the databases MSigDB (Subramanian et al., 2005), GeneSetDB (Araki et al., 2012), and KEGG (Kanehisa et al., 2012) were used.

#### Protein-protein interaction network analysis (PPI)

Using the BioGRID database for *Mus musculus*^4^, we analyzed, for the 4 brain structures independently, the protein–protein interaction (PPI) network of the DEGs. The majority of DEGs for each structure was not annotated, i.e., not associated with a known pathway in BioGRID database (fraction of annotated DEGs: striatum: 71/186; hippocampus: 7/33, cerebellum: 4/24, cortex: 3/22). Cystoscape v3.6.1 was used to visualize the PPI network to find out key genes. A randomized protein interaction network was created using all DEGs to determine the probability of finding a network.

### Quantitative RT-PCR

We selected several genes of interest by cross checking the DEGs, ORA (gene ontology – GO –, Kyoto encyclopaedia of genes and genomes – KEGG– and Pathway), and data from the literature. To validate these genes, we used q-RT-PCR, either with the droplet digital Polymerase Chain Reaction (dd-PCR) or with the real time quantitative PCR (q-PCR) technology.

For dd-PCR, total RNA was extracted as described above and the cDNA library was generated using the iScript advanced cDNA kit (Bio-Rad). The dd-PCR was performed with the dd-PCR supermix for probes (no dUTP, Bio-Rad) and probes labeled with the FAM (for the genes of interest) and HEX (for the *Gapdh* housekeeping gene) fluorophores using the QX100 droplet digital PCR system (Bio-Rad). Results were analyzed using the QuantaSoft Software.

For q-PCR, total RNA (200 ng) was reverse-transcribed with oligo-dT primers using RevertAid First Strand cDNA Synthesis Kit (ThermoFisher). Quantitative PCR was performed in triplicate with a 7,500 real time PCR system (Applied Biosystems) using LightCycler SYBR Green I Master Mix (Roche) and specific pairs of primers. Individual data were normalized using a combination of two housekeeping genes (*Ppia* and *Rpl13a*).

The results are reported as fold changes. All statistical analyses were performed using R software (R Core Team (2020), R Foundation for Statistical Computing). The comparison between genotypes was performed using Mann–Whitney U-tests.

### Single-molecule fluorescent *in situ* hybridization (smFISH) and image analysis

Three month-old mice, deeply anesthetized by an intraperitoneal injection of a mixture of Ketamine (Imalgen®, 200 mg/kg, Merial) and Xylazine (Rompun®, 8 mg/kg, Bayer), were transcardially perfused with 10 ml of PBS, followed by 50 ml 4% paraformaldehyde (PFA) in PBS (Santa Cruz Biotechnology) at 4°C. Each brain was snap frozen in liquid nitrogen and then stored at −80°C. Cryosections (16 μm thick), obtained using the CM300 cryostat (Leica Biosystem) were collected on Superfrost Plus microscope slides (Thermo Fisher Scientific) and stored at −20°C before hybridization. The brain sections (from bregma 1.7 to −0.58) were treated as described by Tsanov et al. (2016) using 48 probes along *Drd1* RNA and 40 probes along *Drd2* RNA (see Supplementary Table S6). Images were acquired in the dorso-medial striatum and in the nucleus accumbens core regions using an Axio observer Z1 inverted microscope (Carl Zeiss) equipped with a Plan-Apochromat 20X/0.8 M27 objective (Carl Zeiss). Images were manually analyzed using imageJ software (NIH). The statistical analyses were performed using R software (R Core Team (2020), R Foundation for Statistical Computing). The comparison between genotypes was performed using Mann–Whitney *U*-tests.

### Confocal fluorescence microscopy on brain sections

Immunofluorescence analyses on striatum sections were performed on 1 year old (52–70 weeks) *Shank3*^∆11^ and 20–28 weeks old *Shank3*^∆4–22^ male mice. Animals, deeply anesthetized as described above, were transcardially perfused with 20 ml of PBS, followed by 50 ml 4% paraformaldehyde (PFA) in PBS (Santa Cruz Biotechnology). Brains were removed and fixed overnight at 4°C in 4% PFA, rapidly washed in PBS, and then immersed in 15% sucrose in PBS for overnight incubation at 4°C. The 15% sucrose solution was then replaced by a 30% sucrose solution for a second overnight incubation at 4°C. Brains were then transferred in Shandon Cryomatrix™ Frozen Embedding Medium (Thermo Scientific™) in cryomolds and frozen by immersion in 2-methyl butane (Sigma-Aldrich) chilled in liquid nitrogen. Samples were kept at −80°C until use. Forty μm coronal sections, obtained using the CM3050S cryostat (Leica Biosystems), were rapidly transferred into PBS in 12-well-culture plates. The samples for GAD65 immunostaining were collected every five sections and grouped together in a separate single well and stored in PBS at 4°C until use. The free-floating sections were rinsed three times in PBS (pH 7.4) and then incubated in NH4Cl (Sigma-Aldrich) 50 mM in PBS for 15 min. After three PBS washes (5 min each), sections were incubated for 1 h at room temperature in PBS containing 1% bovine serum albumin (Applichem) and 0.3% Triton X-100 (Sigma-Aldrich) (PBS/BSA/TX) before incubation for ≈ 20 h at room temperature with the following primary antibodies diluted in PBS/BSA/TX: rabbit polyclonal anti-GAD65 (Invitrogen PA5-77983, 1/200), mouse monoclonal anti-GAD65 (Millipore MAB351, 1/500), guinea pig polyclonal anti MOR (Millipore AB5509, 1/100), rabbit polyclonal anti-VGLUT1 (Synaptic Systems 135303, 1/1000), and mouse monoclonal anti-SHANK3 (Santa Cruz Biotechnology sc-377088, 1/1000). This latter antibody is directed against the C-terminal region of most SHANK3 isoforms. After three PBS washes (10 min each), sections were incubated in secondary antibodies (Alexa fluor 555™-conjugated goat anti-rabbit IgG, Alexa fluor488™-conjugated goat anti-mouse IgG and Alexa fluor488™-conjugated goat anti-guinea-pig IgG (all from Invitrogen)) diluted 1/500 in PBS/BSA/TX for 1 h at room temperature, washed in PBS (3 washes of 10 min) and mounted in FluorSave™ Reagent (Calbiochem). To stain nuclei, a 10 min incubation step in DAPI (1 μg/ml in H_2_0, Thermoscientific) followed by a PBS wash was added before mounting. Images were acquired using a LSM 700 confocal microscope (Carl Zeiss) equipped with a Plan-Apochromat 10X/0.45 M27 objective (Carl Zeiss). For each animal, 5 to 7 coronal sections spanning the anterior–posterior axis from Bregma 1.10 mm to Bregma 0.14 mm were imaged bilaterally. Acquisition parameters were adjusted for each brain hemi-section in order to have no saturating signal for GAD65 in the striatal region of interest. Images of the dorsal striatum were reconstructed by stitching multiple maximum-intensity projected *z*-stacks.

### Quantitative analysis of GAD65 immunoreactivity

To deal with sample to sample variations in labeling efficiency inherent to immunofluorescent labeling methods we determined, for each image, the ratio of the labeling intensities of the striosome and matrix compartments to the adjacent cortex, in which *Gad2* is not differentially expressed in Shank*3*^∆11/∆11^ and *Shank3*^+/+^ mice, according to the transcriptome analysis. For each image, the regions of interest (dorsal striatum and adjacent cortex) were delimited using the Icy software (Institut Pasteur, Paris). In case of edge effect along the lateral ventricle, the concerned edge was excluded from the region of interest. We developed a method to automatically detect three areas in the striatum: the unlabeled myelinated fibers, the matrix (lower expression of GAD65), and the patches/striosomes (higher expression of GAD65). To deal with possible acquisition artifacts, we first applied a mean filter on the image by using a box blur (size 7 × 7). We then pre-computed two maps corresponding to the local mean and standard deviation of the image. For each point, we computed those values considering a window of 1000 × 1000 pixels centered on the point, with a computation step of 10px. Then, for each pixel of the smoothed image, we labeled as fibers all points of intensity value below localMean-localStd. The final fiber mask was eroded morphologically (2px) to remove spurious segmentations and connectivities. To compute the patches, we performed the same computation, but the pixel intensity had to be over localMean+localStd and below localMean + localStd*5 to remove super-bright spots corresponding to fluorescent dust. We also eroded the result by a factor of 2 pixels. Fiber and Patch masks were then processed with a 2D 8-way connected component process to get all individual fibers and patches. We then cleaned up the detection by removing patches of a surface inferior to 100 pixels, and fibers of a surface inferior to 50 pixels. To determine the intensity ratio of matrix versus cortex, we created a matrix segmentation corresponding to the striatal region of interest subtracted by the patches and the fibers detected. The meanMatrix was computed over this surface while the meanPatch was the mean intensity of all pixels contained in the patch mask. For each image, we computed the ratio meanPatch over cortex and meanMatrix over cortex. We also computed the surface of the patch mask relative to the surface of the (patch mask + matrix mask). To determine the effect of genotype on the data extracted by the image analysis, we applied a linear mixed model (LMM) using the method “restricted maximum likelihood (REML)” from the Python library “statsmodels.formula.api.mixedlm.” In all presented results, the LMM has converged and a value of *p* indicating if the datasets from the *Shank3*-mutated and *Shank3*^+/+^ mice are significantly different is provided.

## Results

At 3 months of age, *Shank3*^Δ11/Δ11^ mice, from Cohort 1 and 2, displayed typical body weight, anxiety-like behavior levels and working memory, compared to *Shank3*^+/+^ littermates (Supplementary Figure S1). Considering the frequent regression of patients carrying *SHANK3* mutations, we tested whether a phenotypic deterioration occurred in *Shank3*^Δ11/Δ11^ mice. For that purpose, we tested the main impairments observed in the *Shank3*^Δ11^ mouse model over time, at 3, 8, and 12 months of age: exploratory activity, social communication and stereotyped behaviors in mice.

### Hypoactivity and atypical motor abilities in *Shank3*^Δ11/Δ11^ mice

**Figure 1:**
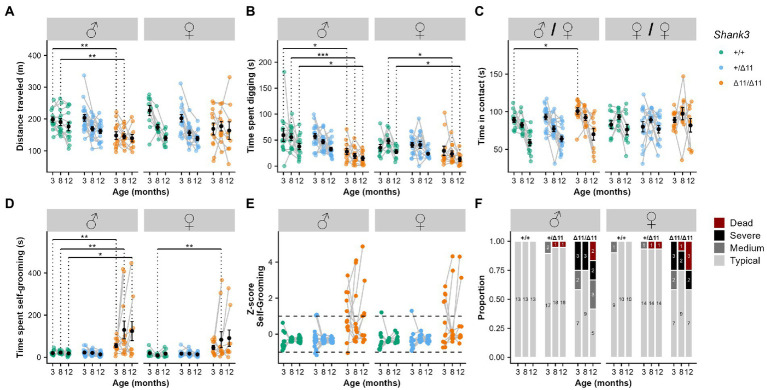
Hypoactivity, reduced exploration and increased stereotyped behaviors in *Shank3*^Δ11/Δ11^ mice at 3, 8, and 12 months of age. **(A)** Total distance traveled during 30-min free exploration of an open field in wild-type (green), heterozygous (blue) and *Shank3*^Δ11/Δ11^ (orange) for males (left panel) and females (right panel). **(B)** Total time spent digging in fresh bedding during 10 min observation in a new test cage, after 10 min habituation. **(C)** Total time spent in contact during male/female and female/female social interaction. **(D)** Total time spent self-grooming during 10 min observation in a new test cage, after 10 min habituation. **(E)** *Z*-score for the time spent self-grooming. **(F)** Proportion and number of individuals displaying different levels of severity in self-grooming. “Dead” corresponds to animals that had to be euthanized due to severe self-inflicted injuries. **(A–D)** Black points represent mean with standard error of the mean (s.e.m). Mann–Whitney *U* test with Bonferroni correction for multiple testing (two tests): * Corrected value of *p* < 0.05; ** corrected value of *p* < 0.01.

Impairment in locomotor activity is often observed in *Shank3* mutant mice (Ferhat et al., 2017). In the open field at 3 months of age, *Shank3*^Δ11/Δ11^ males traveled significantly shorter distances in comparison with *Shank3*^+/+^ littermates (Cohort 1: *W* = 130, *p* = 0.007; Figure 1A, left panel). This observation was not significant for *Shank3*^Δ11/Δ11^ females of Cohort 1 but was confirmed for *Shank3*^Δ11/Δ11^ males of Cohort 2 (Supplementary Figure S2A) and Cohort 3 (Supplementary Figure S2B) at 3 months of age. This reduction of distance traveled in *Shank3*^Δ11/Δ11^ males was not present at older age; however, *Shank3*^+/+^ and *Shank3*^+/Δ11^ mice reduced their activity with increasing age (Figure 1A). A similar trend was present but did not reach significance levels in females (Cohort 1: *W* = 92, *p* = 0.07; Figure 1A). Remarkably, in stressful swimming conditions, *Shank3*^Δ11/Δ11^ mice, from Cohort 3, swam at a significantly higher speed compared to wild-type mice in the water maze with visible platform (Cohort 3: *p* < 0.01 over all training) and in the star maze (*p* < 0.01), without any significant difference to find the direct path in the star maze (Supplementary Figures S2E–G). *Shank3*^Δ11/Δ11^ males also spent less time digging in the bedding in comparison with *Shank3*^+/+^ mice at 3 months (Cohort 1: *W* = 123, *p* = 0.027; Figure 1B; Cohort 2: *W* = 173, *p* < 0.001; Supplementary Figure S2C). Genotype-related differences in digging behavior persisted with increasing age (8 months: *W* = 140, *p* < 0.01; 12 months; *W* = 107; *p* = 0.01; Figure 1B).

### Atypical socio-sexual behavior in *Shank3*^Δ11/Δ11^ male mice at early stage

Impairments in social interactions and communication, some of the core symptoms of autism, were not always observed in the different *Shank3* mouse models (Ferhat et al., 2017). We analyzed the preference for a conspecific during three-chambered test and the time of interaction and types of contact during free dyadic interaction (Supplementary Figure S3). During the three-chambered test, mice of all genotypes and both sexes displayed a typical preference for a same-sex conspecific (Supplementary Figure S4). Furthermore, no significant difference was observed during free dyadic social interaction, at different ages for Cohort 1, in the latency for the first contact (not shown), in the emission of ultrasonic vocalizations (USVs) (Supplementary Figure S4B), and in the total time spent in contact and in the different social behaviors with same-sex conspecific for both sexes (Supplementary Figures S3, S4C–F).

In contrast, we observed that *Shank3*^Δ11/Δ11^ males spent significantly more time in contact with an estrous female compared to *Shank3*^+/+^ males at 3 months of age (*W* = 32, *p* = 0.034; Figure 1C), especially in oro-oral contact (*W* = 22, *p* = 0.005; Supplementary Figures S3, S4G). We also observed a reduction of time keeping the female in the visual field for *Shank3*^Δ11/Δ11^ males compared to *Shank3*^+/+^ males (*W* = 133, *p* = 0.006; Supplementary Figure S4G). These altered behaviors were not accompanied with atypical vocal behavior (Supplementary Figure S4B). At later age, the increase of overall social interaction disappeared during interaction between *Shank3*^Δ11/Δ11^ males and estrous females. However, at 8 months of age, the duration of oro-oral contact in *Shank3*^Δ11/Δ11^ males with an estrous female was still significantly increased (*W* = 29, *p* = 0.025), but these differences were no longer significant at 12 months of age (Supplementary Figures S4H,I). Nevertheless, at this age, we detected an increase of the “approach then escape” behavioral sequence displayed by *Shank3*^Δ11/Δ11^ males toward a C57BL/6 J female in comparison with *Shank3*^+/+^ males (*W* = 15.5, *p* = 0.005, Supplementary Figure S4I).

### *Shank3* mutant mice display excessive self-grooming behavior worsening with age

Self-grooming is a spontaneous and natural behavior displayed by mice for hygienic purposes or in reaction to stressful conditions (Kalueff et al., 2016). Here, we investigated whether this behavior was exacerbated in mutant mice compared to WT littermates in an unfamiliar test environment. At 3 months of age, *Shank3*^Δ11/Δ11^ male mice already displayed a significant increase in the time spent self-grooming in comparison with *Shank3*^+/+^ males for Cohort 1 (*W* = 21, *p* = 0.002) and 2 (*W* = 43, *p* = 0.022). Similar trends did not reach significance in female mice (Figures 1D–F and Supplementary Figure S2D). Furthermore, a subsample of *Shank3*^Δ11/Δ11^ males (5 individuals over 12) and females (6 individuals over 12) presented hair removal that evolved into self-injuries.

At 8 and 12 months of age, Cohort 1 *Shank3*^Δ11/Δ11^ males still displayed increased self-grooming in comparison with *Shank3*^+/+^ mice (8 months: *W* = 27, *p* = 0.009; 12 months: *W* = 37, *p* = 0.041; Figure 1D). The same was true for *Shank3*^Δ11/Δ11^ females who also groomed themselves significantly more than *Shank3*^+/+^ females (8 months: *W* = 11, *p* = 0.002; 12 months: *W* = 20, *p* = 0.014; Figure 1D). With age, an increasing number of *Shank3*^Δ11/Δ11^ mice were outliers, performing self-grooming during a period between one and two standard deviation(s) (medium phenotype) or exceeding two standard deviations (severe phenotype) or had to be euthanized because of severe self-inflicted injuries. Hereafter, we denominate mice with a period of self-grooming over one standard deviation as excessive self-groomers. Nevertheless, for 4 out of 12 *Shank3*^Δ11/Δ11^ males and 3 out of 12 *Shank3*^Δ11/Δ11^ females, we did not observe excessive self-grooming at any of the three time points (Figures 1D,E).

### Differential expression of genes related to synaptic function, signaling and cytoskeleton dynamics in the striatum of *Shank3*^Δ11/Δ11^ mice

To investigate the impact of the loss of the major SHANK3 isoforms on the transcriptome, we performed RNAseq on four brain structures (whole cortex, striatum, hippocampus, and cerebellum) of seven *Shank3*^+/+^ and eight *Shank3*^Δ11/Δ11^ male mice after their behavioral characterization at 12 months of age. We observed a reduction of the transcript levels of the major isoforms of *Shank3* in *Shank3*^Δ11/Δ11^ mice (Supplementary Figure S5). We also observed an enrichment of DEGs in the vicinity of *Shank3* locus on chromosome 15 (before: 10.4 Mb; after: 7.5 Mb) (Supplementary Table S2). This enrichment is most likely due to the residual genomic region from the 129S1/SvImJ ES cells used to generate the *Shank3*^Δ11/Δ11^ mice (Supplementary Figure S6; supplementary information of Schmeisser et al., 2012). We therefore excluded these genes from the analyzes. As expected, *Shank3* expression levels in all four brain structures were significantly reduced in *Shank3*^Δ11/Δ11^ mice in comparison with *Shank3*^+/+^ mice (FDR < 0.001; Supplementary Figure S7). Notably, the *Shank3* sequence reads still present in the *Shank3*^Δ11/Δ11^ mice were aligned to exons downstream of exon 11 (Supplementary Table S2). No significant changes in the expression of *Shank1* and *Shank2* were found (Supplementary Figure S7 and Supplementary Table S2).

**Figure 2:**
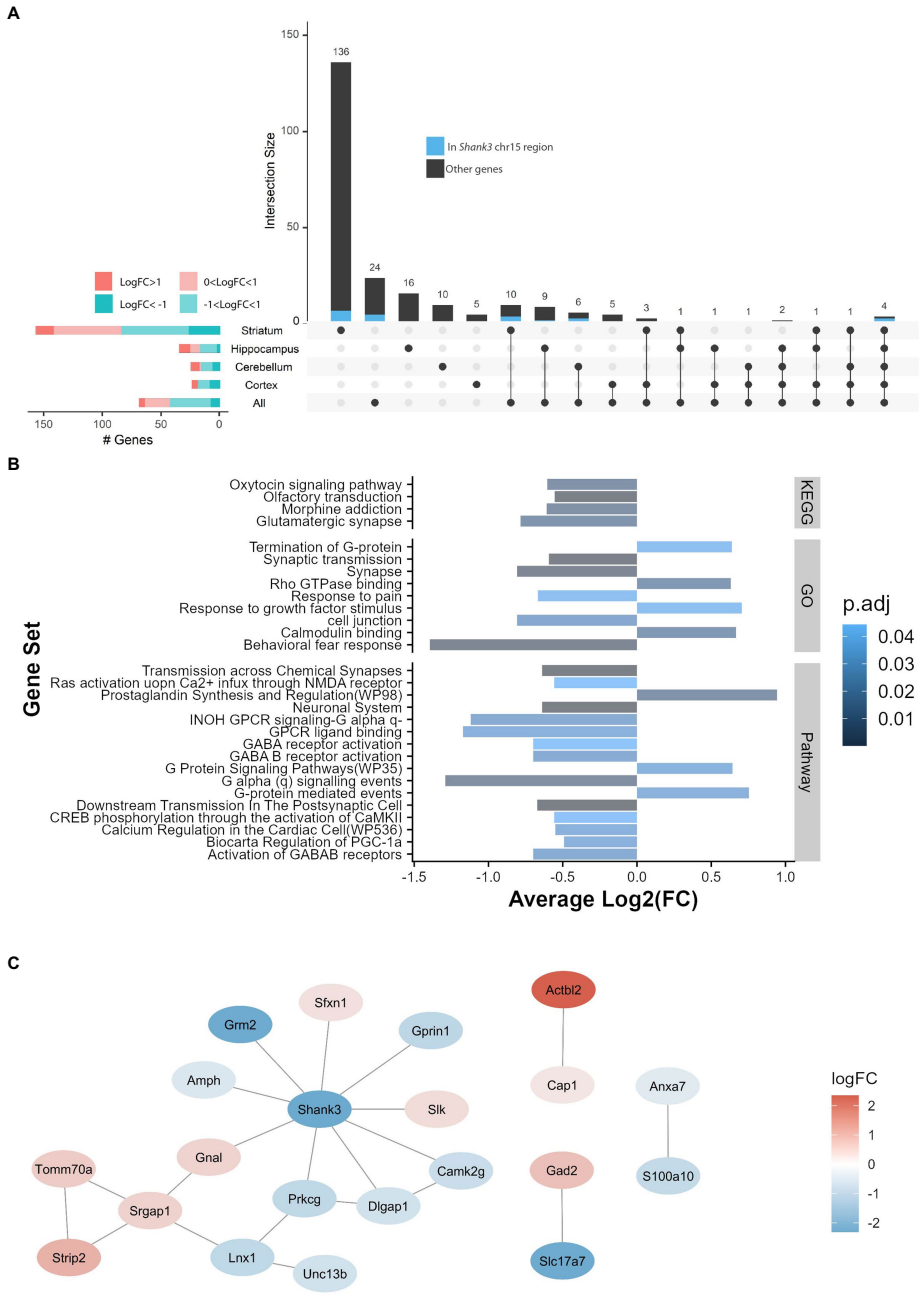
The striatum displays the largest gene expression differences between *Shank3*^+/+^ and *Shank3*^Δ11/Δ11^ mice and the largest number of impacted pathways. **(A)** Upset plot displaying the intersection between brain structures for the genes differentially expressed between *Shank3*^Δ11/Δ11^ and *Shank3*^+/+^ mice (adjusted value of *p* < 0.05; determined by limma-voom using observation quality weights). Genes located around the *Shank3* region on chromosome 15 are shown in blue. The horizontal bar plots on the left show the number of differentially expressed genes in the different brain structures according to the value of their log-fold changes (logFC). **(B)** GO (Gene Ontology), GeneSetDB Pathway (gsdbpath), and KEGG (Kyoto Encyclopaedia of Genes and Genomes) gene sets found to be over-represented (top 20 gene sets with adjusted value of *p* < 0.05) in the genes detected as differentially expressed in the striatum. **(C)** Protein–protein interaction (PPI) network for the differentially expressed genes (DEGs). Nodes, colored ovals, represent DEGs; edges, black lines, represent direct protein–protein interactions (from BioGrid) between DEGs products. The color of the node represents the Log2FC of DEGs: red means up-regulation and blue means down-regulation in *Shank3*^Δ11/Δ11^ mice compared to wild-type mice.

We examined the DEGs (FDR < 0.05) within each of the four brain structures. The largest number of DEGs was detected in the striatum with 140 DEGs. The hippocampus displayed 26 DEGs, the cortex 13 and the cerebellum 16 (Figure 2A). After removing the genes and pseudogenes located around the *Shank3* locus, *Shank3* was the only gene differentially expressed in the four brain structures (Figure 2A). The DEGs in the striatum displayed an increased dispersion compared to the other structures, indicating an increased variability of response between animals (Supplementary Figure S8). Altogether, these results suggest that, among the four structures studied, the transcriptome of the striatum is by far the most impacted by the loss of the major isoforms of SHANK3. We performed quantitative RT-PCR (q-RT-PCR) to confirm up- or downregulation of 30 DEGs (Spearman correlation *R* = 0.809, *p* < 0.0001; Supplementary Figure S9).

We investigated the functions of the proteins encoded by the DEGs in the striatum using in the striatum using ORA and PPI. The ORA (Supplementary Table S3) revealed enrichment in DEGs associated with, among others, two pathways of interest: synaptic transmission (negative fold-change in *Shank3*^Δ11/Δ11^ mice in comparison with *Shank3*^+/+^ mice), and G-protein activity (positive and negative fold-change in *Shank3*^Δ11/Δ11^ mice in comparison with *Shank3*^+/+^ mice according to the type of G-protein pathway) (Figure 2B and Supplementary Table S3). PPI analysis of the striatal DEGs highlighted one network of 15 proteins (value of *p* from randomized network: <0.01; Figure 2C), some of them directly interacting with SHANK3 in the PSD, such as the metabotropic glutamate receptor 2 (encoded by *Grm2*, highly decreased) and Disks large-associated protein 1, a scaffolding protein (encoded by *Dlgap1*, also decreased) interacting with AMPA and NMDA receptors. In addition to these synaptic proteins, the network contains proteins involved in signal transduction (e.g., *Prkcg1*, *Camk2g*) and cytoskeleton dynamics (e.g., *Slk*). Other small networks identified by the PPI analysis contain proteins involved in glutamate transportation (*Slc17a7*) and decarboxylation (*Gad2*), and cytoskeleton dynamics (*Actbl2*).

### Cellular expression pattern of the DEGs expressed in the striatum of *Shank3*^Δ11/Δ11^ mice

**Figure 3:**
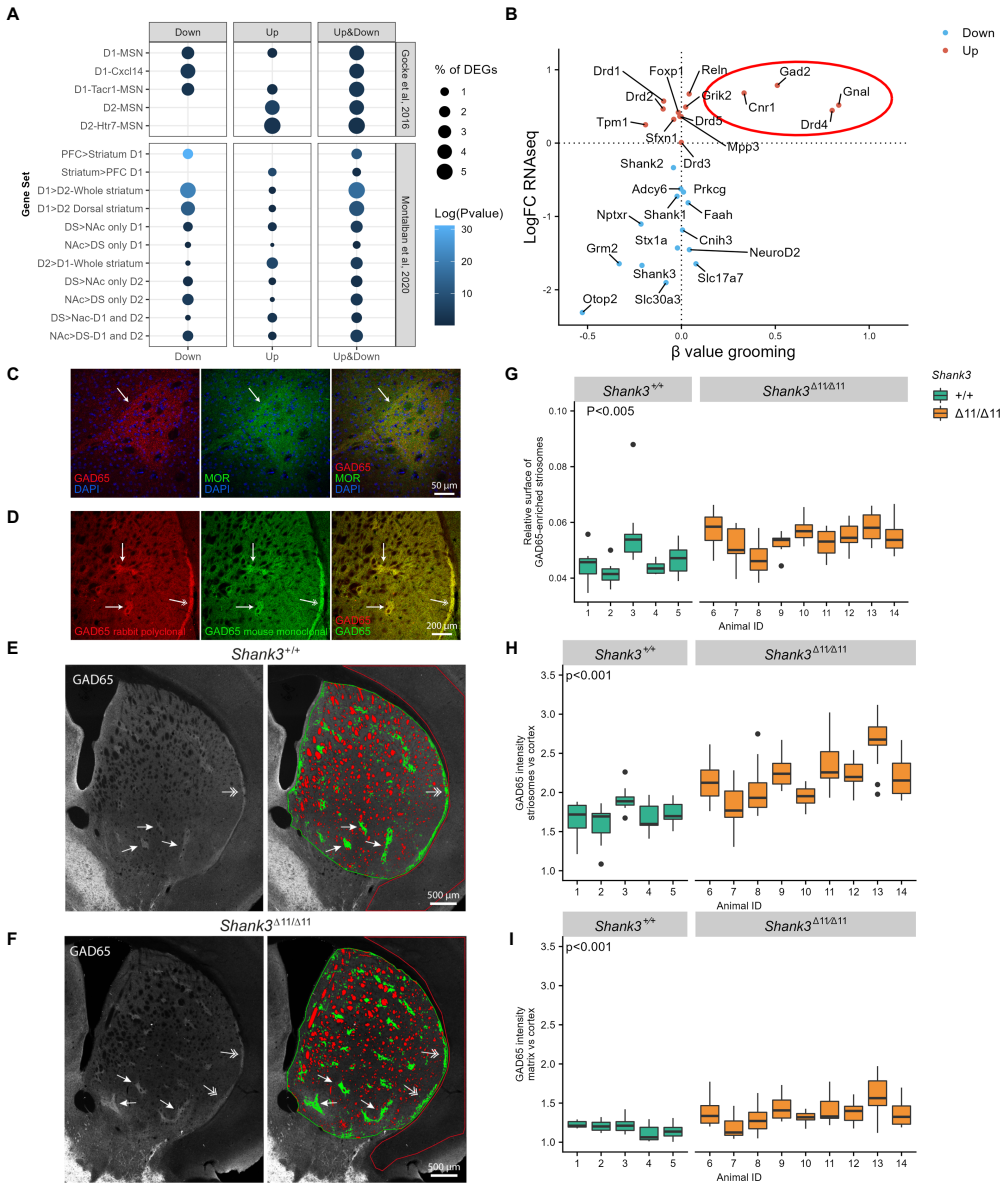
Altered transcriptome of D1- and D2-MSN, over-expression of GAD65, and modification of the striosome/matrix balance in the striatum of *Shank3*^Δ11/Δ11^ mice. **(A)** Enrichment of RNAseq DEGs in the striatum of *Shank3*^Δ11/Δ11^ mice compared to cell-specific gene clusters suggested by Gokce et al. (2016) and Montalban et al. (2020). Genes under-expressed in *Shank3*^Δ11/Δ11^ striatum are enriched in D1-MSN clusters while genes over-expressed in *Shank3*^Δ11/Δ11^ striatum are enriched in D2-MSN clusters. Ratio genes correspond to the proportion of DEGs enriched in the cluster. Over-representation analyses were performed with Fisher’s hypergeometric tests and *p*-values were adjusted for multiple testing with the Benjamini–Hochberg procedure within each category of DEGs. **(B)** Comparison of the log of the fold change of gene expression of selected genes in *Shank3*^Δ11/Δ11^ vs. *Shank3*^+/+^ mice (LogFC RNAseq, *Y* axis) and the *β* values (slope of the linear regression) for low self-grooming vs. high self-grooming (*β* value grooming, *X* axis). **(C,D)** Confocal images of striatum coronal sections of one-year old *Shank3*^+/+^ male mice. **(C)** GAD65 immunoreactivity (red) is enriched in striatal microzones identified as striosomes by immunostaining for μ-opioid receptor (MOR, in green), a canonical marker of striosomes. **(D)** Increased GAD65 immunoreactivity in striosomes (arrows) and subcallosal streak (two-headed arrows) compared to surrounding matrix is observed with two different antibodies: the rabbit polyclonal antibody (Invitrogen PA5-77983 in red) used in **(C,E,F)** and a mouse monoclonal antibody (Millipore MAB351, in green). **(E,F)** Distribution and quantification of GAD65 immunoreactivity in dorsal striatum sections of a *Shank3*^+/+^ **(E)** and a *Shank3*^Δ11/Δ11^ **(F)** mouse (images generated by stitching multiple maximum-intensity projected z-stacks). The arrows point to striosomes and the arrowheads to subcallosal streak. In the dorsal striatum ROI (surrounded in green), the unlabeled myelinated fibers are colored in red, the regions of higher expression of GAD65 (striosomes) are colored in green, and the regions of lower expression of GAD65 (matrix) are uncolored. The cortex ROI used as a reference is surrounded in red. Note that the acquisition parameters were adjusted for each brain hemi-section in order to have no saturating signal for GAD65 in the striatal ROI. Increased intensity in the striatum thus leads to a staining which appears weaker in the cortex. **(G–I)** Comparison of GAD65 immunoreactivity in the striosome and matrix compartments of the dorsal striatum in 5 *Shank3*^+/+^ (green) and 9 *Shank3*^Δ11/Δ11^ (orange) 1-year old male mice. **(G)** Relative surface of the GAD65-enriched striosome compartment (surface of striosomes/surface of (striosomes + matrix). **(H)** Relative GAD65 labeling intensity in the striosomal compartment of the striatum compared to cortex. **(I)** Relative GAD65 labeling intensity in the matrix compartment of the striatum compared to cortex. Data, generated from analysis of 9–14 images per animal, are presented as box-plots (median, first, and third quartiles).

The inhibitory medium-sized spiny neurons (MSN) constitute the major type of striatal neuronal population. They receive glutamatergic inputs and are the target of dopamine innervation from cortex, thalamus, amygdala and substantia nigra pars compacta (SNpc) (Park et al., 2020). Most of these MSN express either dopamine 1 receptor DRD1 (D1-MSN, belonging to the direct pathway), dopamine 2 receptor DRD2 (D2-MSN, belonging to the indirect pathway), or both receptors for a small fraction of MSN. In order to identify the striatal cell types which are the most impacted by SHANK3 deficiency, we compared the expression pattern of the DEGs with marker genes of striatal cell types reported in two single-cell RNA sequencing studies (Gokce et al., 2016; Montalban et al., 2020). We observed that DEGs under-expressed in *Shank3*^Δ11/Δ11^ compared to *Shank3*^+/+^ mice were enriched in the D1-MSN clusters while overexpressed genes were enriched in the D2-MSN clusters (Figure 3A and Supplementary Tables S2, S4). This could reflect a modified proportion of D1-MSN (decrease) and D2-MSN (increase) in the striatum of the *Shank3*^Δ11/Δ11^ mice. However, many genes commonly considered as specific to D1-MSN (*Drd1*, *Isl1*, *Sfxn1*, and *Tac1*) or D2-MSN (*Drd2*, *Adora2a*, *Penk*, *Gpr6*, *Sp9*) were not found to be differentially expressed in the *Shank3*^Δ11/Δ11^ animals (Supplementary Table S2). Moreover, using Single Molecule RNA Fluorescence *In Situ* Hybridization with *Drd1* and *Drd2* RNA probes, we observed no significant difference in the number of D1 and D2 striatal neurons between the *Shank3*^Δ11/Δ11^ and *Shank3*^+/+^ mice (Supplementary Figure S10). Altogether, these results indicate that the deletion of exon 11 of *Shank3* leads to differential transcriptional alterations in the D1- and D2-MSN, without major difference in the number of these types of neurons throughout the whole striatum.

### Abnormal expression of striosome markers

Using RT-PCR, we investigated the correlation between the mRNA levels in the striatum of DEGs identified by RNAseq analysis and other genes of interest (*Shank1*, *Shank2*, *and Drds*) and the level of self-grooming observed in seven 12 months-old *Shank3*^Δ11/Δ11^ mice (Supplementary Figure S11). We found that excessive grooming correlated with higher levels of transcripts for four of the DEGs identified by RNAseq or RT-PCR: *Cnr1*, *Gnal*, *Gad2*, and *Drd4* (Figure 3B). *Cnr1* encodes the cannabinoid receptor 1; *Gnal* encodes a stimulatory G-alpha subunit of a heterotrimeric G-protein (Gαolf); *Gad2* codes for GAD65, one of the two enzymes that convert glutamate into GABA, and *Drd4* encodes the dopamine receptor 4. Interestingly, these proteins have been reported to be heterogeneously distributed in the dorsal striatum, being more abundant in the spatial microzones known as striosomes or patches than in the much larger matrix surrounding them, especially *Gad2/*GAD65 (Rivera et al., 2002; Lévesque et al., 2004; Sako et al., 2010; Davis et al., 2018). Therefore, given the function of GAD65 (i.e., converting glutamate into GABA, an inhibitory transmitter), we studied its distribution in the dorsal striatum, using immunofluorescence on brain sections (Figures 3C–I). Using two different anti-GAD65 antibodies, we found that GAD65 is enriched in striosomes, identified by co-labeling of μ-opioid receptor (MOR), the most widely used marker for striosomes of the rostral striatum (Miyamoto et al., 2018; Figures 3C,D). GAD65 can thus be considered as a marker of the striosomal compartment in mice. Quantitative analysis revealed that the relative surface occupied by the GAD65-enriched striosomes in the dorsal striatum is increased in the *Shank3*^Δ11/Δ11^ mice compared to the *Shank3*^+/+^ mice (*p* = 0.0018, Figures 3E–G). Moreover, in agreement with the overexpression of *Gad2* in the striatum but not in the cortex (Supplementary Table S2) of *Shank3*^Δ11/Δ11^ mice, GAD65 labeling intensity relative to cortex was increased in the whole *Shank3*^Δ11/Δ11^ striatum, but at much higher levels in the striosomes (*p* = 6.5 10^−5^, Figure 3H) than in the matrix (*p* = 2.9 10^−5^, Figure 3I and Supplementary Figures S12A,B). In the striatum of *Shank3*^Δ11/Δ11^ mice, SHANK3 staining is still detected in a small fraction of glutamatergic synapses but the number of puncta and their intensity are highly reduced compared to *Shank3*^+/+^ mice (Supplementary Figures S12A,C). In both *Shank3*^+/+^ and *Shank3*^Δ11/Δ11^ mice, the intensity of the SHANK3 staining is not different between the striosomes and the matrix. Finally, we investigated GAD65 distribution in the striatum of another *Shank3* mouse model, lacking all SHANK3 isoforms (Drapeau et al., 2018). These 20–28 weeks-old *Shank3*^Δ4–22/Δ4-22^ male mice were also used to validate the specificity of the anti-SHANK3 antibody (Supplementary Figure S12). We confirmed enlargement and GAD65 over-expression in the striosomal compartment. However, in the absence of all SHANK3 isoforms, GAD65 is also markedly over-expressed in the matrix (Supplementary Figure S12).

## Discussion

The present study highlighted robust behavioral deficits in *Shank3*^Δ11/Δ11^ mutant mice, more specifically a reduced activity in the open field, atypical social behavior in males interacting with estrous females, and increased self-grooming compared to wild-type littermates. The majority of *Shank3*^Δ11/Δ11^ mutant mice displayed a tendency to a worsening of the self-grooming phenotype with increasing age for mice showing an early emergence of this trait. At 12 months of age, the striatum had the most impacted transcriptomic profile compared to other brain regions. Further molecular characterizations pointed towards possible imbalances between the striosome and matrix compartments in the dorsal striatum.

### Behavioral profiling of the *Shank3^Δ11/Δ11^* mutant mice

#### Atypical social interaction

Results from the literature have highlighted either reduced duration of social contact and reduced number of USVs emitted [e.g., *Shank3*^∆ex4-22^ (Wang et al., 2016)] or no significant difference in socio-sexual behaviors [e.g., *Shank3*^Q321R^ (Yoo Y.-E. et al., 2019)]. Here, we observed limited difference in social interaction with an increase in the time spent in contact for *Shank3*^Δ11/Δ11^ males interacting with an oestrous female (observed at 3 and 8 months of age), and more specifically in nose-to-nose contacts, as already highlighted in previous studies [*Shank3*∆^ex14 − 16^cKO (Yoo T. et al., 2019); *Shank3*^Δ11/Δ11^ females (de Chaumont et al., 2021)]. Testing the specificity of this type of contact would help to understand its significance in mouse social communication. Further experiments should be designed to test whether this specific behavior is associated with a deficit in social reward processing [as suggested for *Shank2*^Δ6–7/Δ6–7^ mice (Ey et al., 2018)]. Indeed, several studies have shown that SHANK3 controls maturation of social circuits in the ventral tegmental area (VTA) (Bariselli et al., 2016; Bariselli and Bellone, 2017) and synaptic strength of D2-MSN in the striatum (Wang et al., 2017; Bey et al., 2018).

#### Excessive grooming

Excessive self-grooming has been observed in the majority of *Shank3* mutant mice over their lifetime and therefore represents the most robust behavioral phenotype in all models. Extreme self-grooming is most likely not due to increased skin sensitivity since rescuing normal tactile reactivity in *Shank3b*^+/−^ mice does not improve over-grooming behavior (Orefice et al., 2019). In our study, we also observed an important inter-individual variability in this trait with 30% of the mutant mice that did not seem to display excessive grooming even after 1 year. Increased self-grooming might reflect a response to stressful conditions, which might vary from one individual to another (difference in susceptibility and/or exposure to stressful conditions). Such increased reactivity to novel or stressful conditions is reminiscent of what is observed in patients and deserves further systematic testing in the different *Shank3* mutant models (Krüttner et al., 2022).

### From transcriptome to imbalance of striatum compartments and excessive self-grooming

#### Abnormal striatal transcriptome

The massive impact of the *Shank3*^Δ11^ mutation on the transcriptome profile of the striatum suggests that this region could be the centerpiece of the behavioral deficits observed in *Shank3*^Δ11/∆11^ mutant mice. Several studies have already reported functional alterations of the striatum and more specifically of the MSN in the diverse *Shank3* mutant strains (Peça et al., 2011; Bariselli et al., 2016; Peixoto et al., 2016; Bariselli and Bellone, 2017; Wang et al., 2017; Bey et al., 2018). Our report of an alteration of the striatal transcriptome could be due to a direct role of the SHANK3 protein in gene transcription. Indeed, a previous study, using transfected cells expressing EGFP-SHANK3 fusion proteins, has reported the presence of SHANK3 in the nucleus, especially the isoform SHANK3b (Wang et al., 2014), which is disrupted in our model. Using immunohistofluorescence, we did not observe SHANK3 in the nucleus of the striatal cells in *Shank3*^+/+^ adult mice, but the epitope recognized by the antibody we used is absent from the SHANK3b isoform. Alternatively, the abnormal striatal transcriptome profile could be the consequence of the alteration of the glutamatergic synapses that interfere with signal transduction, such as G protein (Qin et al., 2018) and mTOR (Moutin et al., 2021) signalling, and then with gene transcription or mRNA stability. We found that several transcripts encoding proteins involved in glutamatergic synaptic transmission were under-expressed in *Shank3*^∆11/∆11^ mice. For example, we observed a decrease of *Grm2*, encoding mGluR2, a G-protein-coupled glutamate receptor which interacts with the proline-rich domain of SHANK3 and which has been found decreased in a valproate-induced rat model of autism (Chen et al., 2014). We also observed significant under-expression of *Dlgap1*, coding for DAP1/GKAP. This protein connects SHANK3 to the scaffolding protein PSD-95 (Boeckers et al., 1999) which recruits AMPAR and NMDAR. In contrast, we did not observe diminution of transcript levels for several proteins (GluA, GluN, or mGluR5) previously shown as decreased in the synaptosomes or PSD fractions of *Shank3* mutant mice compared to wild type. A possible explanation is that the absence of SHANK3 decreases the translation or the recruitment of these proteins at the synapse or increases their degradation without affecting gene transcription and mRNA degradation. Interestingly, we observed a sexual dimorphism in behavior in mice, especially for cohort 1, that is not reported in patients (Leblond et al., 2014). This aspect could be explained by the impact of estradiol on striatal function and behavior in mice (Meitzen et al., 2018), as well as by differential gene expression between males and females after an acute stress in mice as, for instance, in the CA3 subregion of hippocampus (Marrocco et al., 2017). Analyzing the brain transcriptome of *Shank3*^+/+^ and *Shank3*^∆11/∆11^ females should shed light on the differential effect of the lack of *Shank3* between sexes.

#### A link between abnormal gene transcription, striatum organization and behavioral impairment

We observed that the expression of *Gad2*, *Cnr1*, *Gnal*, and *Drd4* was positively correlated with excessive self-grooming. Remarkably, three of these genes have been previously associated with self-grooming, but not always in the same direction. In contrast to our study, *Cnr1* deletions (as well as CB1R antagonists) have been reported to increase self-grooming behavior (Litvin et al., 2013; Gogolla et al., 2014). The overexpression of *Cnr1* in *Shank3*^∆11/∆11^ mice might therefore result from a compensatory upregulation in response to the alterations of the endocannabinoid system which has been previously reported in SHANK3-deficient mice (Wang et al., 2017; Folkes et al., 2020), in valproic acid induced rat models of autism and also in some autistic individuals (Zou et al., 2021). Consistent with our findings, *Gnal* haploinsufficiency is associated with a reduction of self-grooming behavior in a mouse model of dystonia (Pelosi et al., 2017). Links between *Gad2* and *Shank3* were previously reported with an increase of GAD65 immunoreactivity in the *Shank3*ß^−/−^ mice (Gogolla et al., 2014) and a decrease of *Gad2* transcript in the striatum of a *Shank3*-overexpressing model (Lee et al., 2017). GAD65 is associated with different disorders related to anxiety, such as obsessive–compulsive disorder, panic disorder or generalized anxiety disorder (Karunakaran et al., 2021). To our knowledge, there is no direct evidence in the literature for an association between *Drd4* expression and grooming behavior. However, *Drd4* is a candidate gene for obsessive–compulsive disorder and panic disorder (Taj et al., 2013). It would thus be interesting in future studies to investigate this potential link.

Interestingly, these four genes have also been reported to be enriched in striosomes (Rivera et al., 2002; Lévesque et al., 2004; Sako et al., 2010; Davis et al., 2018). These microzones of the dorsal striatum contain early born MSN and are embedded into the much larger surrounding matrix including late-born MSN (Hagimoto et al., 2017). The two compartments, initially distinguished by differential expression of numerous molecular markers [for a review see (Crittenden and Graybiel, 2011)], also differ in their input and output connectivity and electrophysiological characteristics (Prager and Plotkin, 2019), such as D1-MSN excitability (Prager et al., 2020), rates of dopamine release (Jaquins-Gerstl et al., 2021), and response to chronic stress (Friedman et al., 2017). Imbalances in striosome to matrix activity have been suggested in several movement disorders [Huntington’s disease, Parkinson’s disease, dyskinesia, dystonia, for a review see (Crittenden and Graybiel, 2011)], psychostimulant-induced motor stereotypies (Canales and Graybiel, 2000), and more recently in anxiety disorder (Karunakaran et al., 2021). Corticostriatal path targeting striosomes also control decision-making under cost–benefit conflict (Friedman et al., 2015, 2020) and habit formation (Nadel et al., 2021). Our finding that four striosome markers had increased expression associated with excessive self-grooming in *Shank3*^Δ11/Δ11^ mice prompted us to explore the compartmental architecture of the striatum in these mice. This investigation was also motivated by previous studies that have shown that enhanced striosomal activation is highly correlated with increased repetitive behaviors, including self-grooming in both non-human primates and rodents [for review see (Kalueff et al., 2016)]. Studies by Kuo and Liu (2017, 2020) have recently suggested that aberrant striatal compartmentation may be involved in autism. Furthermore, through the investigation of protein–protein interactions, a role for the striosomes in anxiety disorder such as social anxiety has been recently proposed (Karunakaran et al., 2021).

We first confirmed that GAD65, which had been reported to be a striosome marker in primates, but not in rats (Lévesque et al., 2004) was indeed a striosome marker in the mouse. We then found that the GAD65-enriched striosomal compartment is enlarged in *Shank3*^Δ11/Δ11^ mice compared to *Shank3^+/+^* mice and that striatal GAD65 overexpression in the absence of the major SHANK3 isoforms is much more pronounced in the striosomes than in the matrix. Whereas the majority of GABA in brain is produced by the cytosolic isoform of glutamate decarboxylase (GAD67, encoded by *Gad1*), GAD65, which is anchored to the membrane of synaptic vesicles, can supply GABA in situations of high demand (Kaufman et al., 1991), such as stress or fear, and for fine tuning of inhibitory transmission. It is thus possible that the altered compartmentation and/or GAD65 over-expression in the striatum are responsible for some of the behavioral features of the *Shank3*^Δ11/Δ11^ mice, especially excessive self-grooming. Future studies, notably by modulating GAD65 expression in the dorsal striatum of wild-type and *Shank3* mice, are needed to establish causality between the striatal alterations and the excessive self-grooming of *Shank3* mice. Moreover, testing, at the molecular and cellular levels in the striatum and at the behavioral level, the impact of stress in *Shank3* mice may help understand the inter-individual variability in the severity of excessive self-grooming.

#### A model for striosomes/matrix imbalance as a possible cause of excessive grooming in the *Shank3^∆11/∆11^* mice

**Figure 4:**
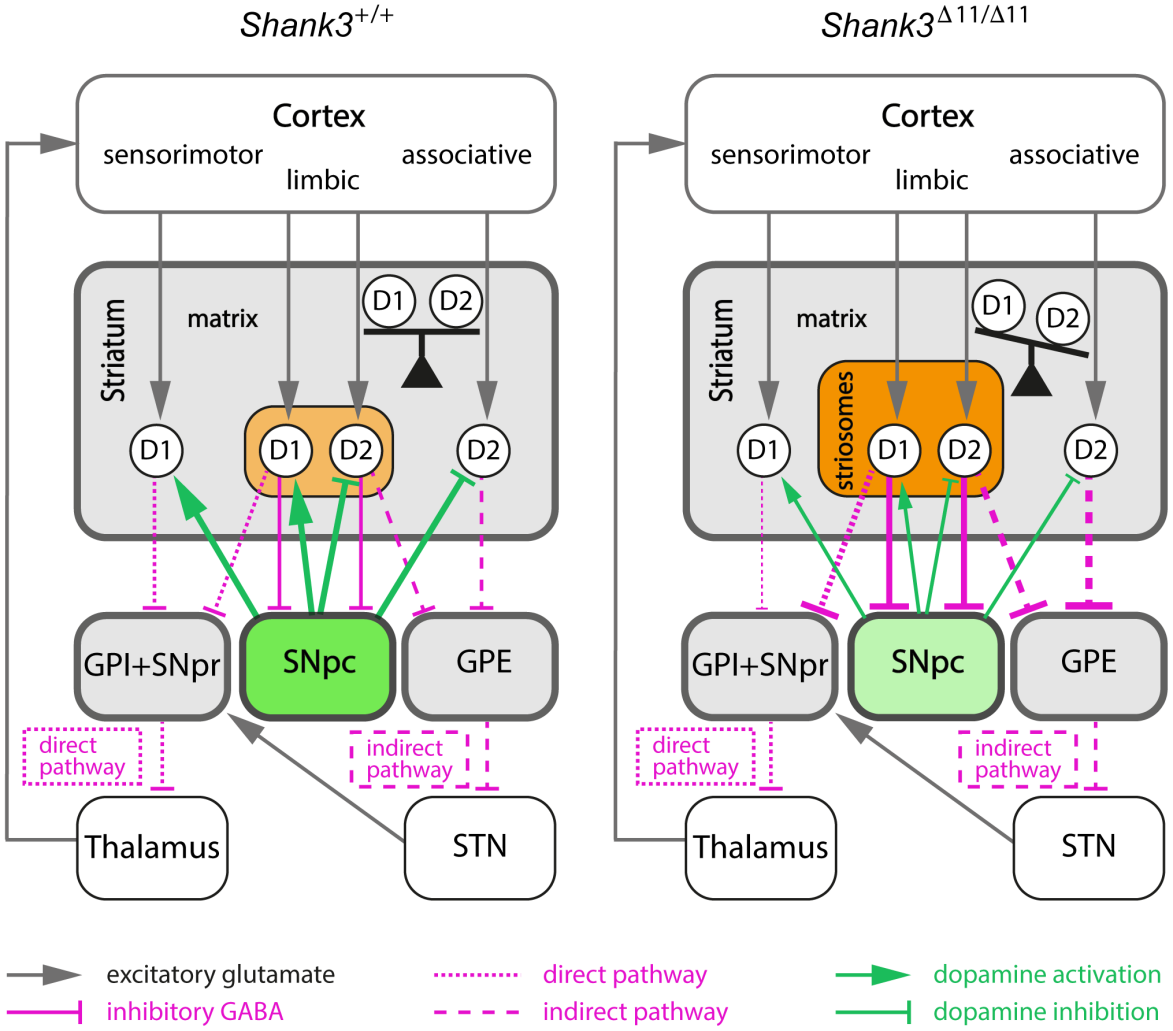
Proposed model linking increased activity in the striosomal pathway and imbalance between the direct and indirect pathways in the *Shank3*^Δ11/Δ11^ mice. The modifications of connection strength (line thickness) induced by striosomal compartment alterations in *Shank3*^Δ11/Δ11^ mice are only represented between the striatum and its output target areas (colored boxes). Increased activity of the striosomal pathway in *Shank3*^Δ11/Δ11^ mice, due to both larger size and increased GAD65 activity of the striosomal compartment (light/dark orange) enhances GABAergic inhibition of the dopamine-producing neurons of SNpc (green). This inhibition of the SNpc neurons (light green) results in an imbalance between the direct and indirect pathways by decreasing both the activation of D1-MSN (D1) and the inhibition of D2-MSN (D2). GPI, internal globus pallidus; GPE, external globus pallidus; SNpr, substantia nigra pars reticulata; SNpc, substantia nigra pars compacta; STN, subthalamic nucleus.

It is well established that the striatum and the dopamine-containing nigrostriatal tract control self-grooming behavior (Kalueff et al., 2016), and that striosomal MSN are the predominant striatal population innervating dopamine neurons of the nigrostriatal tract (Watabe-Uchida et al., 2012). An imbalance between the striosome and the matrix compartments may then explain, *via* a modification of dopamine signalling, the excessive grooming of *Shank3*^Δ11/Δ11^ mice (Figure 4). Indeed, in contrast to MSN located in the matrix of the striatum, striosomal MSN projects directly to the dopamine-producing neurons in the SNpc (Crittenden and Graybiel, 2011; McGregor et al., 2019). Then, SNpc dopamine neurons project back to the entire dorsal striatum. An increased activity of GAD65 in striosomes, in a situation of stress for example, would lead to an inhibition of dopamine release by the SNpc and thus to a reduced dopamine modulation of both the direct and indirect pathways in the dorsal striatum. Because dopamine activation has opposite effects on D1- and D2-MSN by increasing and decreasing cell excitability, respectively, a decreased dopamine release by SNpc is expected to result in diminishing D1-MSN activity and boosting D2-MSN activity. The differential enrichment in up- and down-regulated DEGs in D2-MSN and D1-MSN, respectively, could be related to previous observation of an impairment of long-term depression in D2-MSN, but not in D1-MSN (Wang et al., 2017) in *Shank3* mutant mice. Importantly, striosome cells are generated before matrix cells during development (Hagimoto et al., 2017) and SHANK3 seems to be involved in the early stages of neuronal development (Peixoto et al., 2016; Jaramillo et al., 2020). Therefore, the enlargement of the total relative surface of the GAD65-enriched striosomal compartment observed first in the *Shank3*^Δ11/Δ11^ and then in the *Shank3*^Δ4–22/Δ4-22^ mice may be due to a default in the compartmentation of the striatum during early development. A future longitudinal study, at the cellular and molecular levels, of striatal development during the late embryonic and early postnatal stages in *Shank3* mice should help determine whether SHANK3 is involved in one of the sequential steps (generation of D1- and D2-MSN, cell migration, segregation of striosome and matrix cells) of striosome/matrix mosaic organisation in the developing striatum.

## Conclusion and perspectives

Altogether, the present study describes the effects over time of the *Shank3*^Δ11^ mutation on mouse social and repetitive behaviors and suggests a critical role of the striatum in excessive self-grooming, probably through imbalances in two intertwined systems, the striosome/matrix compartments and the direct/indirect pathways. Our results suggest that SHANK3 deficiency/Phelan-McDermid syndrome and possibly to some extent autism could integrate the growing list of neurological conditions implicating an imbalance in the striosome/matrix compartmentation of the dorsal striatum. Future studies in mice, but also in other species such as rat (Harony-Nicolas et al., 2017) or non-human primates (Zhao et al., 2017; Zhou et al., 2019), are necessary to establish causality between the here-reported striatal defects and the stereotyped behaviors of *Shank3* mice, to understand the mechanism at the origin of the striosome/matrix imbalance and finally consider the possibility of its reversibility.

## Data availability statement

The datasets presented in this study can be found in online repositories. The names of the repository/repositories and accession number(s) can be found in the article/Supplementary material.

## Ethics statement

The animal study was reviewed and approved by the ethical committees of Institut Pasteur (CEEA n°89) and Sorbonne Université (CEEA n°5).

## Author contributions

ATF, ABi, EV, EE, and TB conceived the experiments. ATF and EE designed and acquired behavioral data from cohorts 1 and 2. ATF extracted and dissected brains, extracted RNA from cohorts 1 and 2, acquired the data from smFISH, performed the analysis and statistics of behavior, PPI and smFISH. ATF and FM designed the smFISH experiments. ABo, BFi, and JFD performed the RNA sequencing. ABi and ATF performed the analyses of ORA. ATF, BFo, and AL performed and analyzed the results of quantitative RT-PCR. EV performed the immunofluorescence experiments, with the help of SC for brain collection. FdC performed quantitative image analysis. SC and AMLS genotyped the mice. JS, CR, and LRR conducted the experiments on cohort 3. TMB generated the *Shank3*^Δ11/Δ11^ mouse model. ATF, ABi, EV, BFo, FdC, EE, and TB wrote the manuscript. All authors contributed to the article and approved the submitted version.

## Funding

This research was supported by Institut Pasteur, the Bettencourt-Schueller Foundation, CNRS, Université de Paris, the Conny-Maeva Charitable Foundation, the the Fondation Cognacq-Jay, the Fondation de France, the Fondation pour la Recherche Médicale (FRM), the Association Française du Syndrome Phelan-McDermid, the Association Téhani et les enfants Phelan-McDermid, the GIS “Autisme et Troubles du Neuro-Développement,” the Roger de Spoelberch Foundation, the Eranet-Neuron (ALTRUISM) project, the Laboratory of Excellence GENMED (Medical Genomics) [grant no. ANR-10-LABX-0013 managed by the National Research Agency (ANR) part of the Investment for the Future program], the BioPsy Labex (ANR-10-LABX-BioPsy, ANR-11-IDEX-0004-02), AIMS-2-TRIALS which received support from the Innovative Medicines Initiative 2 Joint Undertaking under grant agreement No 777394 and the Inception program (Investissement d’Avenir grant ANR-16-CONV-0005). We gratefully acknowledge the UtechS Photonic Bioimaging (Imagopole), C2RT, Institut Pasteur, supported by the French National Research Agency (France BioImaging; ANR-10-INSB-04; Investments for the future).

## Conflict of interest

The authors declare that the research was conducted in the absence of any commercial or financial relationships that could be construed as a potential conflict of interest.

The reviewer MY declared a shared affiliation with the author ATF to the handling editor at the time of review.

## Publisher’s note

All claims expressed in this article are solely those of the authors and do not necessarily represent those of their affiliated organizations, or those of the publisher, the editors and the reviewers. Any product that may be evaluated in this article, or claim that may be made by its manufacturer, is not guaranteed or endorsed by the publisher.

## Author disclaimer

The views expressed here are the responsibility of the authors only. The EU Commission takes no responsibility for any use made of the information set out.

## Footnotes

^1^ https://ftp.ebi.ac.uk/pub/databases/mousegenomes/REL-1505-SNPs_Indels/mgp.v5.merged.snps_all.dbSNP142.vcf.gz

^2^ https://software.broadinstitute.org/gatk/guid#e/article?id=3891

^3^ http://broadinstitute.github.io/picard

^4^ https://thebiogrid.org/

## Abbreviations

dd-PCR: droplet digital Polymerase Chain Reaction
DEGs: differentially expressed genes
DRD1/2: D1/D2 dopamine receptor
D1/2-MSN: medium-sized spiny neurons expressing the D1/D2 receptor
GABA: γ-aminobutyric acid
ORA: over-representation analysis
PPI: protein–protein interaction
PSD: postsynaptic density
q-PCR: real time quantitative PCR
RNAseq: RNA sequencing
SNP: single nucleotide polymorphism
SNpc: substantia nigra pars compacta
USV: ultrasonic vocalisations
