# Supplementary material for: Excessive self-grooming, gene dysregulation and imbalance between the striosome and matrix compartments in the striatum of Shank3 mutant mice
Source: Front Mol Neurosci. 2023 Mar 16;16:1139118. doi: 10.3389/fnmol.2023.1139118 (PMC10061084; doi:10.3389/fnmol.2023.1139118)
Supplement: Supplementary file 7 [file Data_Sheet_1.pdf]

Supplementary Figures

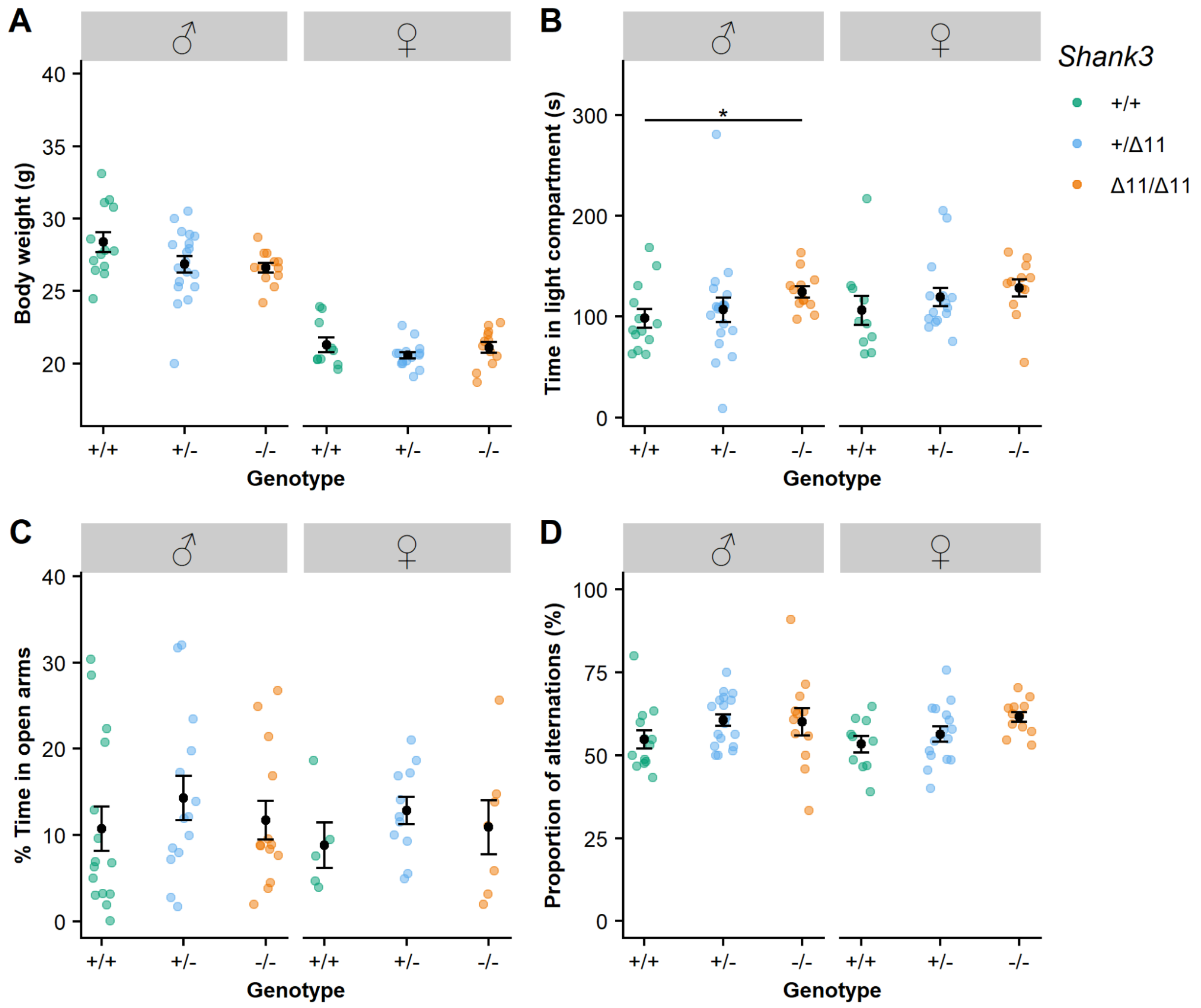

Supplementary Figure S1

Three-month-old *Shank3*$^{\Delta11/\Delta11}$ mice display subtle genotype-related differences with their *Shank3*$^{+/+}$ and *Shank3*$^{+/\Delta11}$ littermates in weight, motor coordination, anxiety and working memory. (A) Body weight of male (left panel) and female (right panel) of Cohort 1. (B) Time spent in the light compartment in the dark/light test during 5 min for Cohort 1. (C) Proportion of time spent in the open arms in the elevated plus maze during 10 min for Cohort 2. (D) Proportion of alternations between the three arms of a Y-maze for Cohort 1. Mann–Whitney U test with Bonferroni correction for multiple testing: *: corrected p-value <0.05; data are presented as mean ± s.e.m. (black squares and circles); 12–18 mice per group.

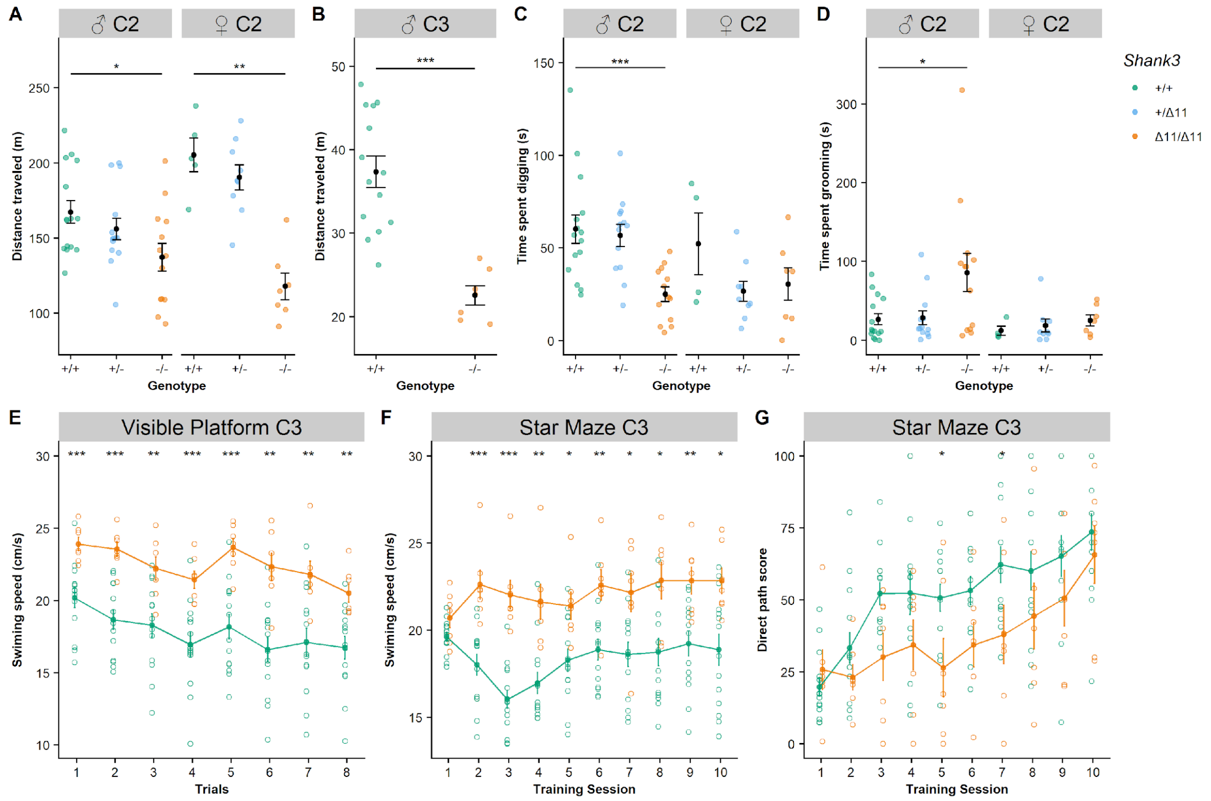

Supplementary Figure S2

Shank3$^{\Delta11/\Delta11}$ mice of Cohorts 2 (C2) and 3 (C3) at three months of age display atypical activity and exploration and increased stereotyped behavior. (A, B) Total distance travelled during free exploration of an open-field for Cohort 2 (C2) during 30 min (A) and Cohort 3 (C3) during 10min (B). (C) Total time spent digging, i.e. moving the bedding with front and/or hind legs, during 10 min observation in a test cage (after 10 min habituation). (D) Total time spent self-grooming during 10 min observation in a test cage with fresh bedding (after 10 min habituation). (E) Swimming speed in a water maze with a visible platform. (F) Swimming speed in a starmaze. (G) Direct-path score in the Starmaze test. Mann–Whitney U test, with Bonferroni correction for multiple testing (in black): *corrected p-value <0.05, **corrected p-value <0.01, ***corrected p-value <0.001; data are presented as mean ± s.e.m.; cohort 2: 8–22 mice per group; cohort 3: 7–14 mice per group.

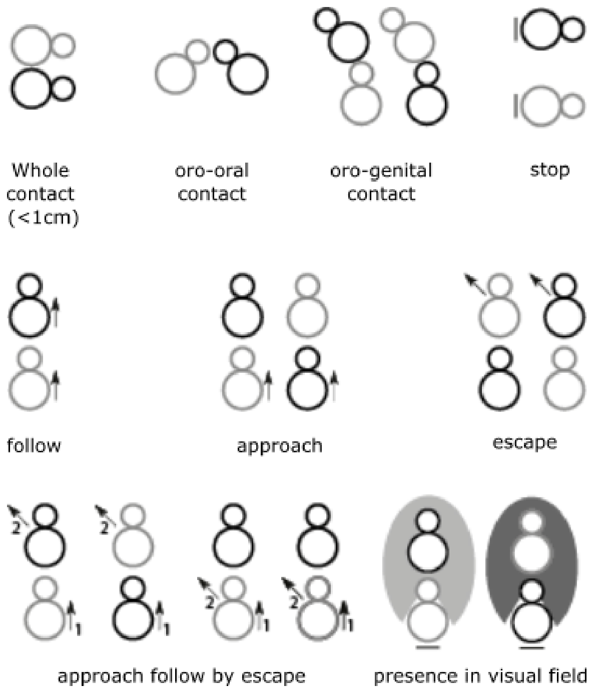

Supplementary Figure S3

Behavioral events detected by Mice Profiler during social interactions between the occupant (light grey) and the new-comer (dark grey). Arrow defines the movement of one of the animals.

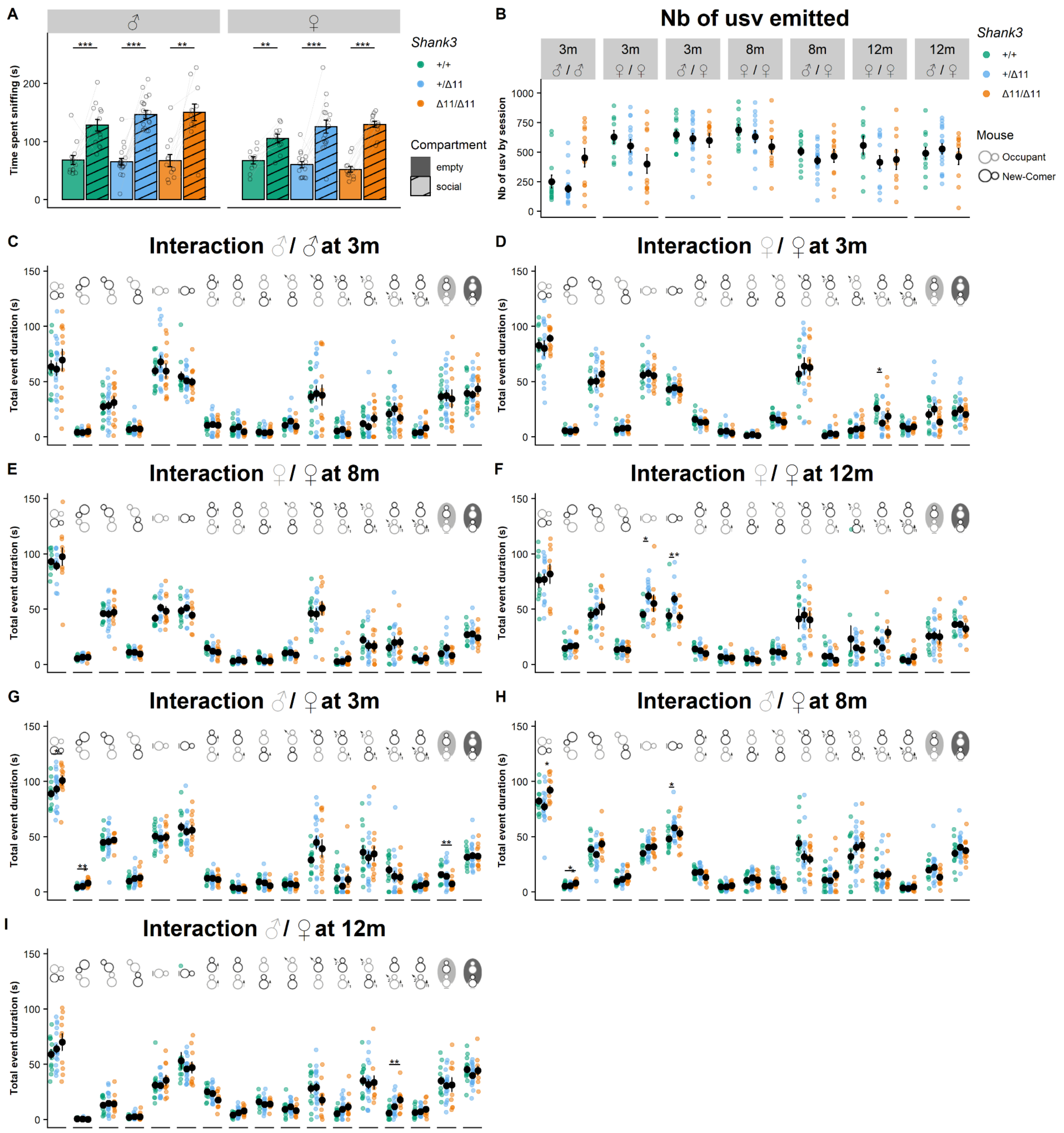

Supplementary Figure S4

Shank3$^{\Delta 11/\Delta 11}$ mice display limited genotype-related differences in social events at three, eight and twelve months of age. (A) Social preference measured in time spent sniffing during the first phase of a three-chambered test. (B) Number of ultrasonic vocalisations (USVs) emitted during 4-min dyadic social interaction. (C – I) Time spent in the different types of social events during 4-min male/male (C), male/female (E, G, I) and female/female (D, F, H) interactions. In all free moving tests, the tested mouse (the occupant) is indicated in light grey and interacts with a wild-type C57BL/6J mouse (the new-comer) indicated in dark grey. Mann–Whitney U test, with Bonferroni correction for multiple testing (in black): *corrected p-value <0.05, **corrected p-value <0.01, ***corrected p-value <0.001; data are presented as mean ± s.e.m.; 12–14 mice per group.

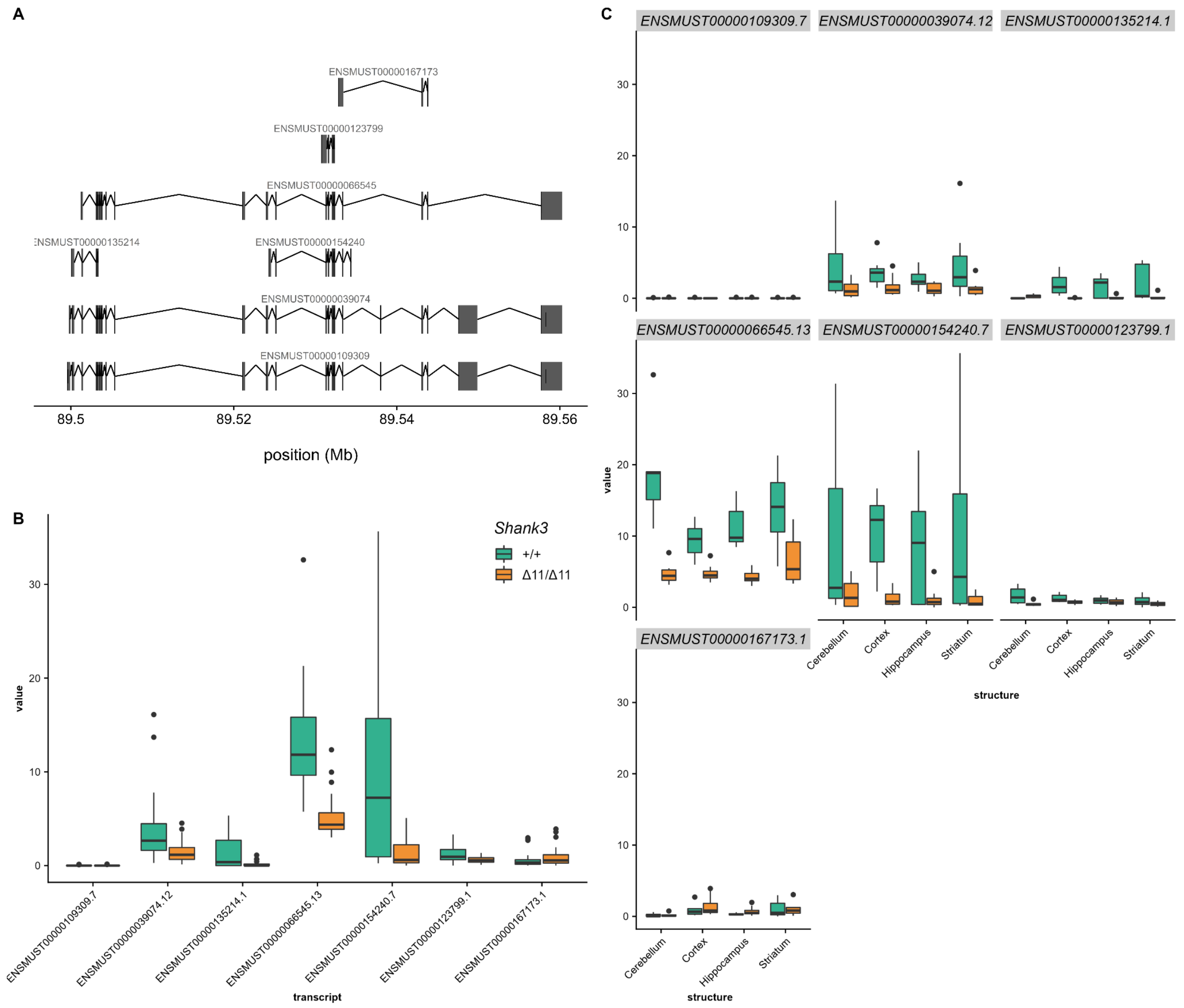

Supplementary Figure S5

Shank3 isoform abundance estimates were computed with Salmon (Patro et al., 2017) using the *Mus Musculus* GRCm38/mm10 genome and Ensembl transcripts annotations. Isoform abundances are measured in log2 transcripts-per million (TPM). (A) *Shank3* Ensembl isoforms. (B) Box plots of *Shank3* isoform abundances (log2 TPM) across all samples in *Shank3*$^{+/+}$ (green) and *Shank3*$^{\Delta11/\Delta11}$ (orange) mice. (C) Box plots of *Shank3* isoform abundances (log2 TPM) in *Shank3*$^{+/+}$ (green) and *Shank3*$^{\Delta11/\Delta11}$ (orange) mice within each brain structure (cerebellum, cortex, hippocampus and striatum).

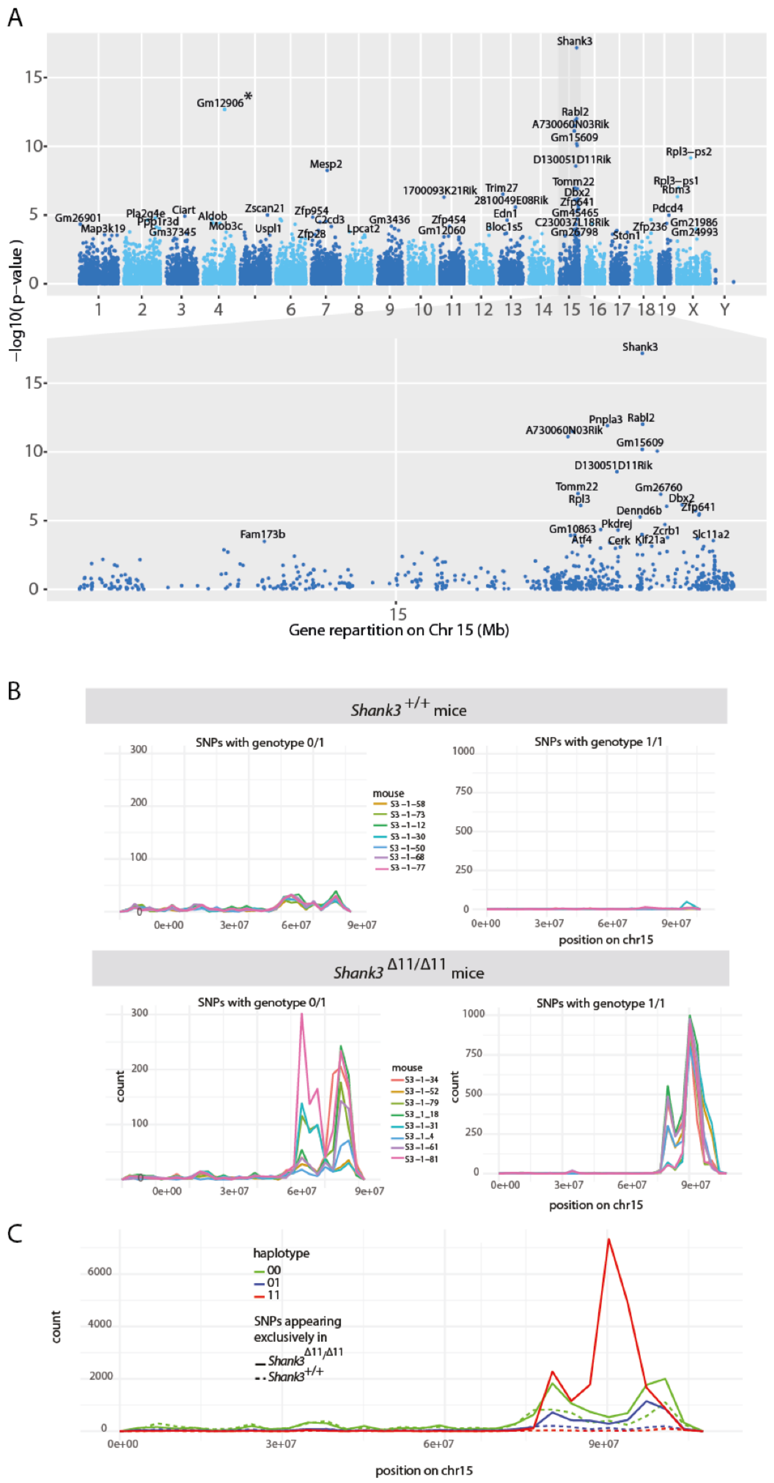

Supplementary Figure S6

An increased number of SNP (single nucleotide polymorphisms) around the *Shank3* locus suggests a construct bias. In the region spanning between 10.4 Mb upstream and 7.5 Mb downstream of *Shank3*, SNPs differing from the reference genome of C57BL/6J mice were found in *Shank3*$^{Δ11/Δ11}$ mice, but not in *Shank3*$^{+/+}$ mice. (A) Manhattan plot showing the negative log10-transformed p-value of the comparison (across all brain structures) of gene expression between *Shank3*$^{+/+}$ and *Shank3*$^{Δ11/Δ11}$ mice for each read on the whole genome (upper panel) and a close-up view on the chromosome 15 (lower panel). The star (*) indicates

the *Gm12906* gene, a pseudogene of the *Tomm22* gene located close to *Shank3* on chromosome 15. (B) Distribution on chromosome 15 of the heterozygous variant genotypes (0/1; right panel) and homozygous variant genotypes (1/1; left panel) that are specific to either *Shank3*$^{+/+}$ (upper panels) or *Shank3*$^{\Delta 11/\Delta 11}$ mice (lower panels). Data are presented as the counts of variants as a function of the genomic position on chromosome 15; each line represents an individual, with samples from all brain regions pooled together. (C) Distribution of the homozygous and heterozygous variants across individuals of the same genotype. Data are presented as the counts of variants as a function of the genomic position on chromosome 15. Homozygous (resp. heterozygous) variant genotypes are shown in red (resp. blue). Plain (resp. dotted) lines represent variants found in *Shank3*$^{\Delta 11/\Delta 11}$ (resp. *Shank3*$^{+/+}$) mice.

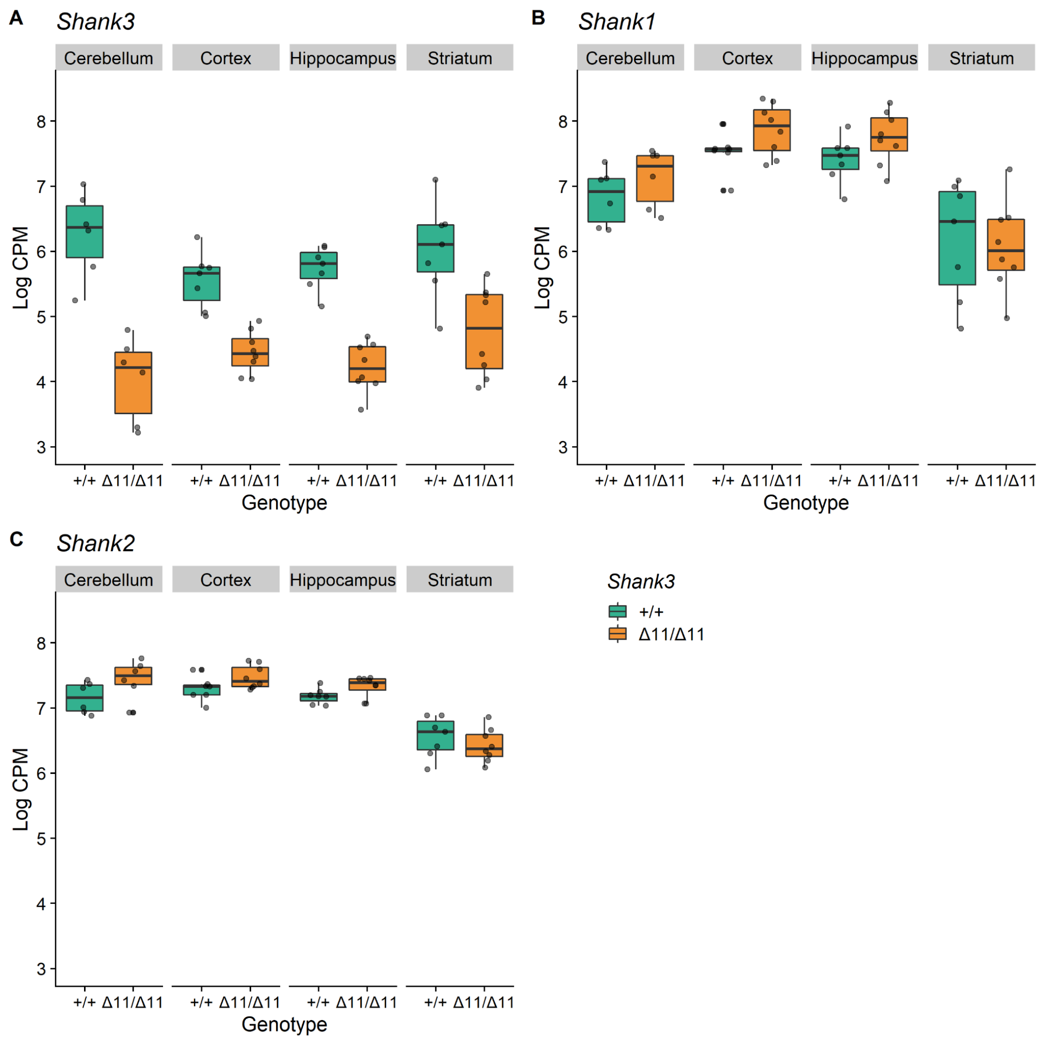

Supplementary Figure S7

RNA sequencing-based expression levels of *Shank1*, *Shank2* and *Shank3* in four brain regions. Log counts of reads per million (RPM) for *Shank3* (A), *Shank1* (B) and *Shank2* (C) transcripts in the cerebellum, cortex, hippocampus and striatum for *Shank3*$^{+/+}$ (green) and *Shank3*$^{\Delta11/\Delta11}$ (orange) mice. Data are presented as box-plots (median and first and third quartiles) and sample points (black points).

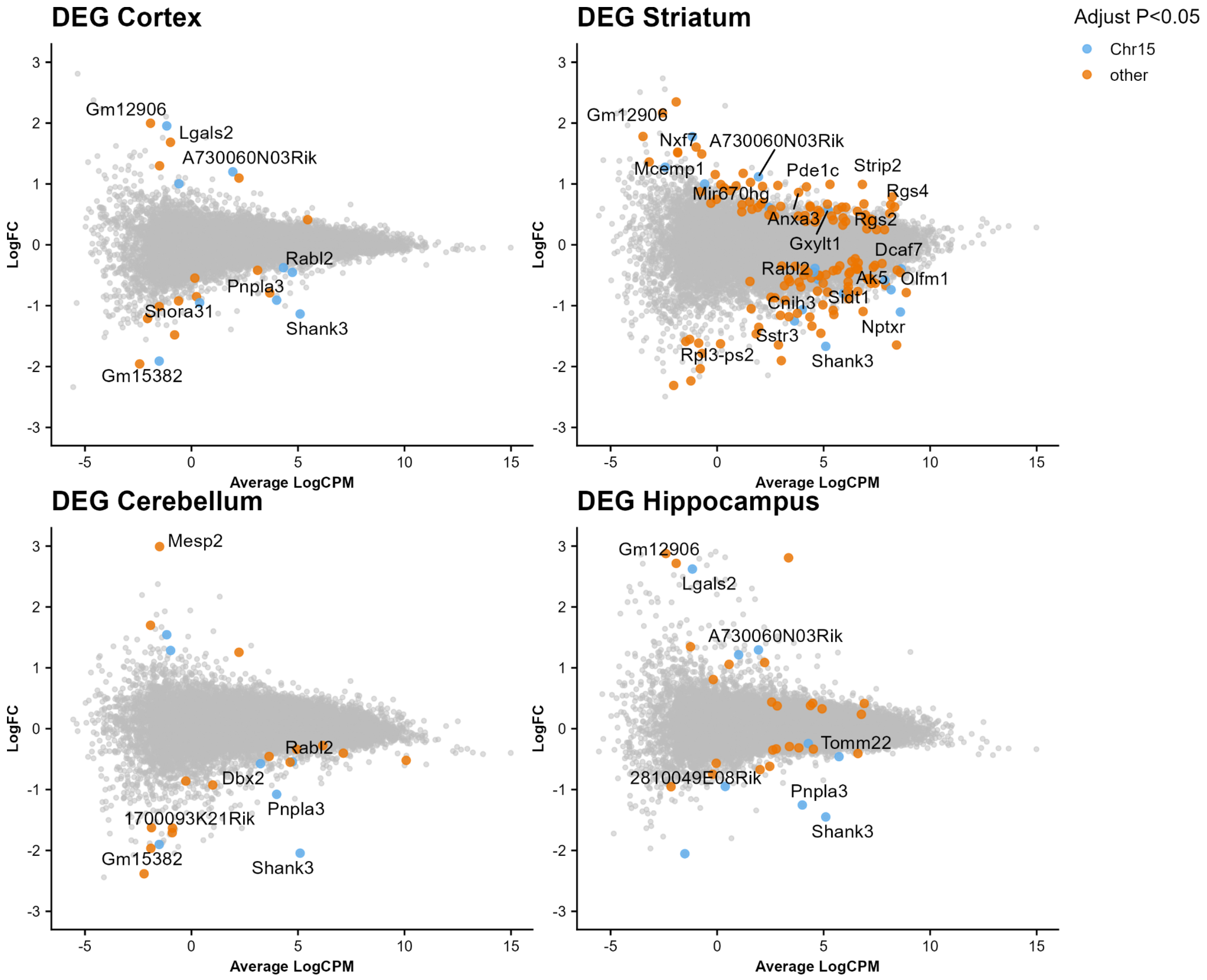

Supplementary Figure S8

Gene expression differences in four brain regions of *Shank3*$^{\Delta11/\Delta11}$ mice compared to *Shank3*$^{+/+}$ mice. Mean-difference plots showing $\log_2$FC between *Shank3*$^{\Delta11/\Delta11}$ and *Shank3*$^{+/+}$ samples as a function of logCPM in the cortex (upper left), striatum (upper right), cerebellum (lower left) and hippocampus (lower right). Genes with a FDR lower than 5% are indicated by a coloured dot, blue for the ones located in the *Shank3* region on chromosome 15, and orange for the others. The star (*) indicates a pseudogene of a gene close to *Shank3* on chromosome 15. Only the names of the genes with an absolute log fold-change value larger than 1 are indicated.

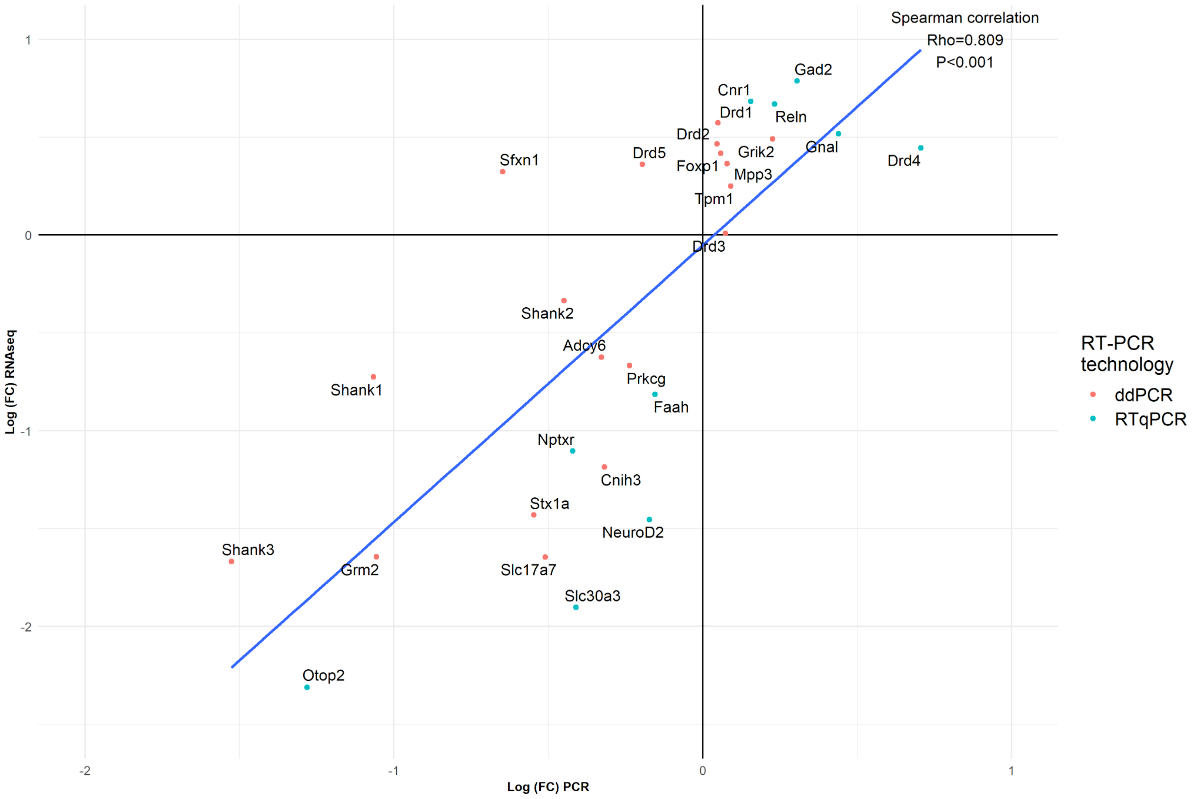

Supplementary Figure S9

Validation by quantitative RT-PCR of DEGs identified by RNAseq analysis in the striatum. Differential striatal expression between *Shank3*$^{+/+}$ and *Shank3*$^{\Delta 11/\Delta 11}$ mice of genes selected among the DEGs identified by RNA sequencing, as well as *Shank1*, *Shank2, and Drds* genes, using either the qRT-PCR (in blue) or dd-PCR (in red) technology.

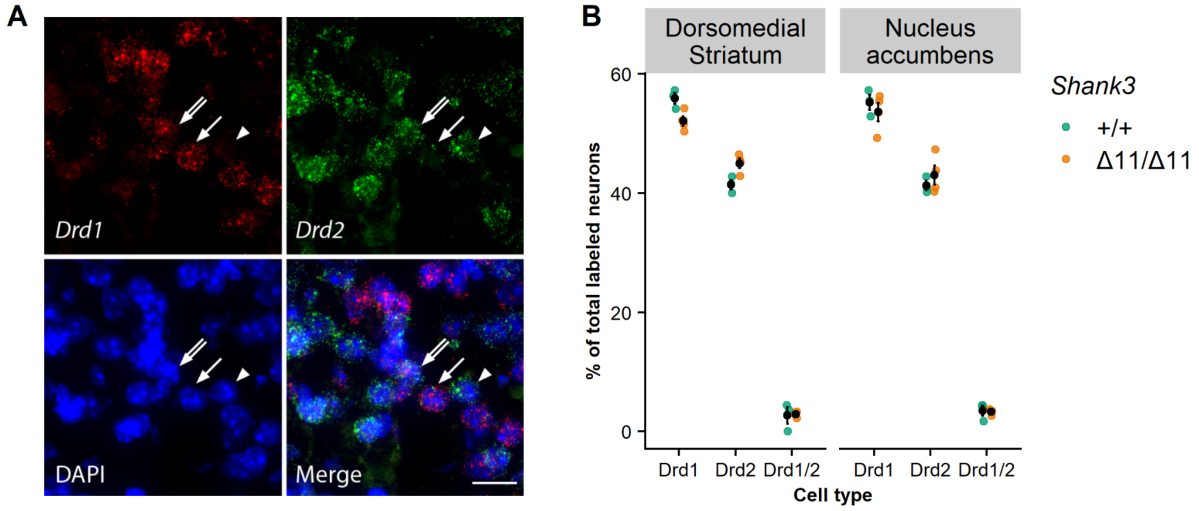

Supplementary Figure S10

The proportion of D1-MSN and D2-MSN is not affected in the *Shank3*$^{\Delta11/\Delta11}$ mice. (A) smFISH staining of D1 (*Drd1*, red) and D2 (*Drd2*, green) dopamine receptors transcripts. Nuclei are stained by DAPI (blue). Bright points represent RNA staining. White arrows point to the cell body of a D1-MSN, arrowheads to the cell body of a D2-MSN, and double arrows to the cell body of a D1/D2-MSN. (B). Proportion of nuclei associated with the *Drd1* or/and *Drd2* staining in the dorsomedial striatum and in the nucleus accumbens core in *Shank3*$^{+/+}$ mice (green) and *Shank3*$^{\Delta11/\Delta11}$ mice (orange). Green and orange points represent individuals (10-15 images per individual) and black points are means ± s.e.m.

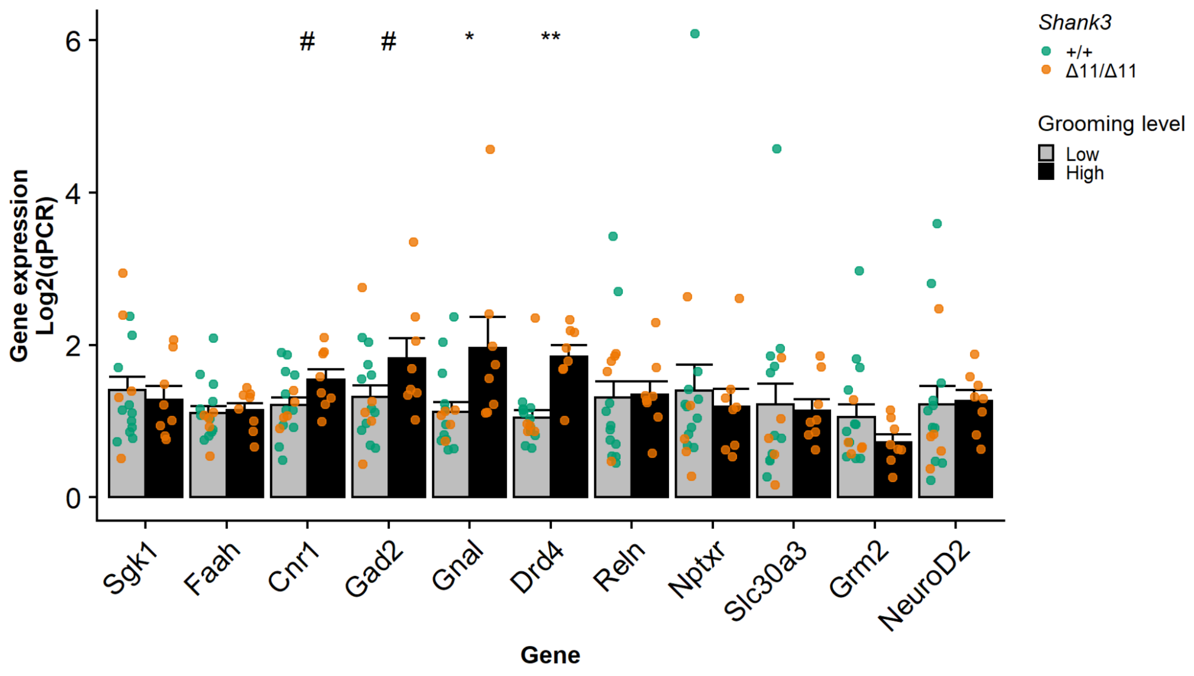

Supplementary Figure S11

Gene expression in mice with low or high self-grooming. Striatal expression of genes (RT-q-PCR) selected among the DEGs identified by RNA sequencing in mice with low or high self-grooming. Mann–Whitney U test: #: $p<0.1$; *: $p<0.05$; **: $p<0.01$.

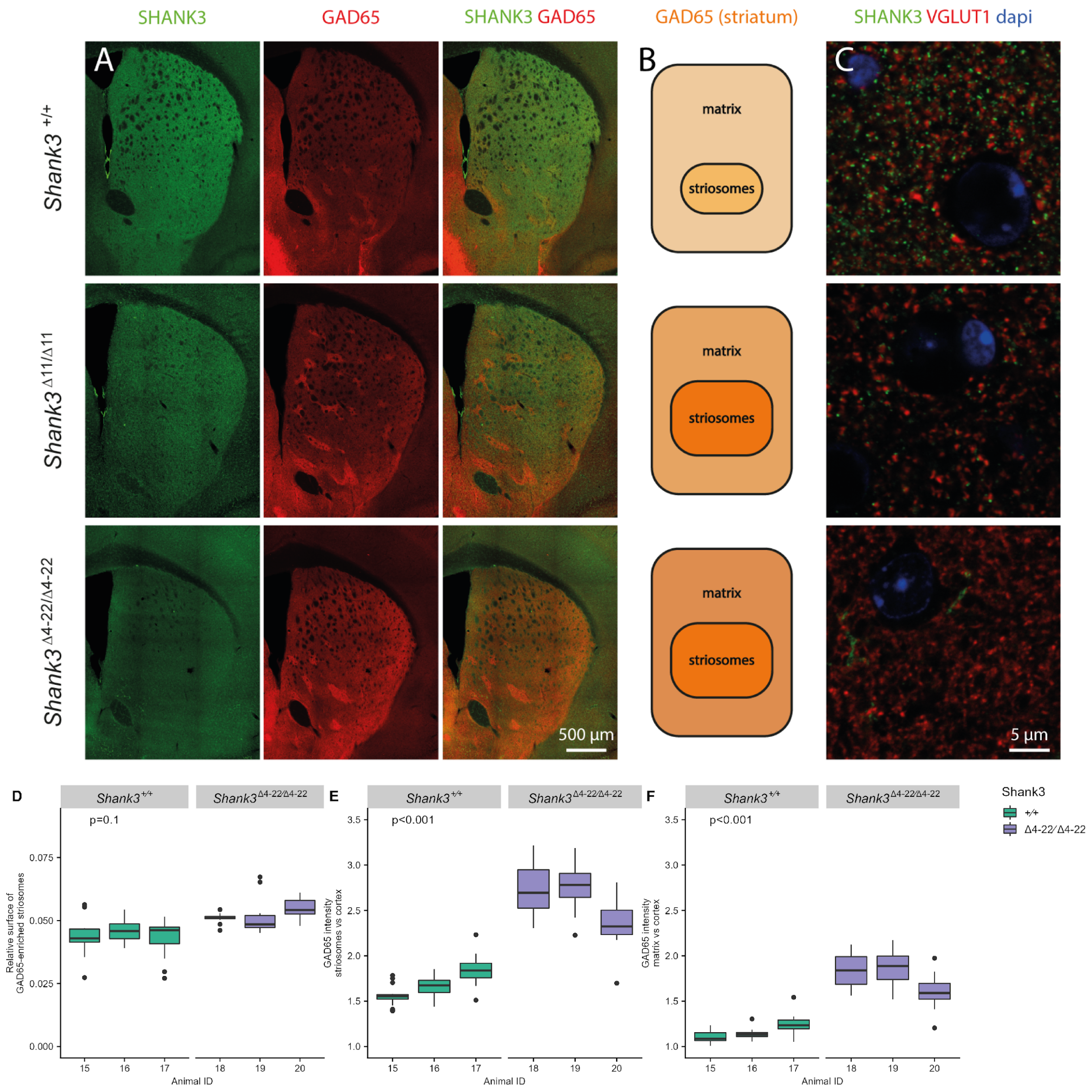

Supplementary Figure S12

Over-expression of GAD65 and enlargement of the striosomal compartment in the striatum of two different SHANK3-deficient mouse models, *Shank3*$^{\Delta11}$ and *Shank3*$^{\Delta4\text{-}22}$. (A) SHANK3 and GAD65 in the striatum. Images were generated by stitching multiple confocal images of coronal brain sections immunostained for SHANK3 and GAD65 (green in left panels and red in centre panels, respectively, merged images in right panels) in *Shank3*$^{+/+}$ (upper panels), *Shank3*$^{\Delta11/\Delta11}$ (middle panels) and *Shank3*$^{\Delta4\text{-}22/\Delta4\text{-}22}$ (lower panels) mice. Note that SHANK3 immunoreactivity is comparable in striosomes and matrix. (B) Schematic representation of GAD65 over-expression (colour gradient from light to dark orange) and of the enlargement of the striosomal compartment in the striatum of the *Shank3*$^{\Delta11/\Delta11}$ (middle panel) and the *Shank3*$^{\Delta4\text{-}22/\Delta4\text{-}22}$ (lower panel) mice, compared to *Shank3*$^{+/+}$ mice (upper panel). GAD65 overexpression is much greater in the striosomes than in the matrix in *Shank3*$^{\Delta11/\Delta11}$ mice that still express some SHANK3 isoforms, while in the *Shank3*$^{\Delta4\text{-}22/\Delta4\text{-}22}$ complete knock-out mice, GAD65 over-expression is great in both compartments. (C) Specificity of the anti-SHANK3

antibody. As expected for a protein of the post-synaptic density of the glutamatergic synapse, in the *Shank3*$^{+/+}$ mice (upper panel), SHANK3-positive puncta (green) are adjacent to the red puncta revealing VGLUT1, a marker of the pre-synaptic density of the glutamatergic synapse. A residual specific staining is still observed in the *Shank3*$^{\Delta11/\Delta11}$ mice (middle panel), while no synaptic puncta is observed in the *Shank3*$^{\Delta4\text{-}22/\Delta4\text{-}22}$ mice (lower panel). Nuclei are stained in blue by DAPI. (D, E, F) Comparison of GAD65 immunoreactivity in the striosome and matrix compartments of the dorsal striatum in 3 *Shank3*$^{+/+}$ (green) and 3 *Shank3*$^{\Delta4\text{-}22/\Delta4\text{-}22}$ (orange) 20-28 weeks old male mice. (D) Relative surface of the GAD65-enriched striosome compartment (surface of striosomes / surface of (striosomes + matrix). (E) Relative GAD65 labelling intensity in the striosomal compartment of the striatum compared to the cortex. (F) Relative GAD65 labelling intensity in the matrix compartment of the striatum compared to the cortex. Data, generated from analysis of at least 11 images per animal, are presented as box-plots (median, first, and third quartiles).

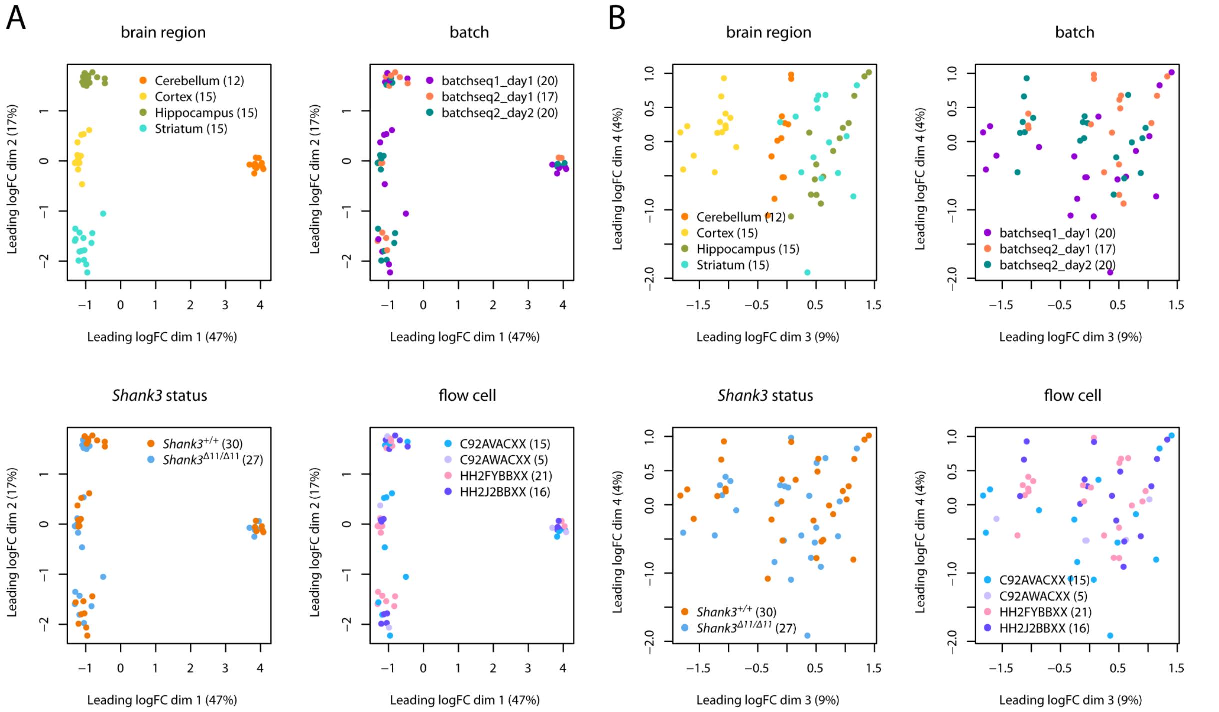

Supplementary Figure S13

Multidimensional scaling (MDS) plots of the gene log counts-per-million (logCPM). (A) Data showing the positions of the samples in the space spanned by the first and second MDS dimensions. (B) Data showing the positions of the samples in the space spanned by the third and fourth MDS dimensions. In (A) and (B), samples are coloured with respect to brain structure (upper left), batch of sequencing and RNA extraction (upper right), genotype (lower left), and flow cell (lower right).

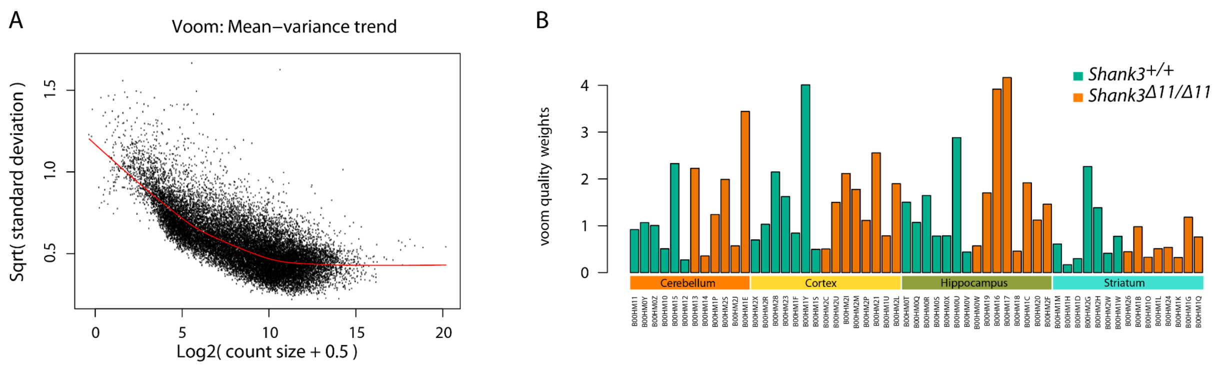

Supplementary Figure S14

Mean-variance relationship. (A) Voom mean-variance trend output for the RNAseq data. Gene-wise means and variances of the RNAseq data are represented by black points with a LOWESS trend. (B) 'Voom' sample-specific weights. Each bar plot represents the weight of one sample used to model variability between different samples.

Supplementary reference

Patro, R., Duggal, G., Love, M.I., Irizarry, R.A., and Kingsford, C. (2017). Salmon provides fast and bias-aware quantification of transcript expression. Nat Methods 14(4), 417-419. doi: 10.1038/nmeth.4197.
